# Are protein substitutes available in Italy for infants with inherited metabolic diseases all the same?

**DOI:** 10.3389/fnut.2025.1581295

**Published:** 2025-05-26

**Authors:** Margherita Di Costanzo, Martina Tosi, Martina Muzi, Enrico Vito Buono, Valentina D’Onghia, Anna Munerati, Gabriele Tarditi, Simone Bruni, Elvira Verduci, Giacomo Biasucci

**Affiliations:** ^1^Pediatrics and Neonatology Unit, Guglielmo da Saliceto Hospital, Piacenza, Italy; ^2^Department of Medicine and Surgery, University of Parma, Parma, Italy; ^3^Department of Health Sciences, University of Milan, Milan, Italy; ^4^Department of Pediatrics, Vittore Buzzi Children’s Hospital, Milan, Italy; ^5^Pediatric Clinic, Department of Medicine and Surgery, University of Parma, Parma, Italy; ^6^Metabolic Diseases Unit, Department of Pediatrics, Vittore Buzzi Children’s Hospital, Milan, Italy

**Keywords:** inherited metabolic diseases, infant nutrition, protein substitutes, tyrosinemia, maple syrup urine disease, glutaric aciduria type 1, classic homocystinuria, organic acidemias

## Abstract

**Introduction:**

Inherited metabolic diseases (IMDs) represent a major clinical challenge, especially during the neonatal and infant periods. They require tailored and long-term nutritional management to ensure proper growth and development. Protein substitutes are essential in the dietary treatment of IMDs, particularly aminoacidopathies, organic acidemias, and urea cycle disorders. In Italy, a variety of PSs is available for infants with IMDs requiring a controlled protein and/or amino acid intake; however, differences in their nutritional composition may impact clinical outcomes. This study aims to examine and compare the nutritional composition of infant PSs (IPSs) available on the Italian market, focusing on macronutrients, micronutrients, and functional components.

**Methods:**

The analysis targets products used in the dietary management of aminoacidopathies, organic acidemias, and urea cycle disorders during the first year of life. We compared the nutritional composition of products intended for healthy infants, considering the Commission Delegated Regulation (EU) 2016/127 and Commission Delegated Regulation (EU) 2016/128. Phenylketonuria is excluded from this analysis, as it has been recently addressed in another paper.

**Results:**

For each condition, there are only two products available, except for isovaleric aciduria and urea cycle disorders, which have only one product. The results indicate higher energy, linoleic, and alpha-linolenic acid content (+9%, +55%, and +290% compared to the maximum reference value), and lower levels of lactose, vitamin D, choline, selenium, and iodine (−92%, −34%, −37%, −12%, and −39% compared to the minimum reference value) for several IPSs. The analysis revealed the presence of docosahexaenoic acid (DHA), and eicosapentaenoic acid (EPA) in all IPSs, while half of them contain arachidonic acid (ARA).

**Discussion:**

This study represents the first comprehensive comparison of the nutritional profiles of IPSs for IMDs on the Italian market. The results identify potential areas for optimization, aiming to provide adequate levels of micronutrients, essential fatty acids, and functional ingredients, such as biotics, to support gut health, immune function, and neurodevelopment.

## 1 Introduction

Inherited metabolic diseases (IMDs) represent a significant clinical challenge, particularly during the neonatal and infant periods. Long-term nutritional management is essential to support optimal growth, development, and metabolic stability. IMDs such as phenylketonuria (PKU), tyrosinemia, maple syrup urine disease (MSUD), glutaric aciduria type 1 (GA1), classic homocystinuria (HCU), isovaleric aciduria (IVA), methylmalonic aciduria (MMA), and propionic aciduria (PA) require infant protein substitutes (IPSs) for their management (1). For urea cycle disorders (UCDs), no IPS is required whereas essential amino acids (EAAs) or branched-chain amino acids (BCAAs) can be used during acute treatment (2). Breast milk, due to its low protein content, is not contraindicated for aminoacidopathies, organic acidemias, and urea cycle disorders, and can be offered to infants with IMDs, in association with specific IPS lacking the toxic AAs (1, 3). Moreover, adequate energy, protein, and AAs intake is necessary to promote anabolism. On the other hand, an excessive protein restriction may lead to metabolic decompensation (1). Breast milk is most compatible with IMDs such as PKU, tyrosinemia, GA1, HCU, MSUD, and IVA, where the amount of breast milk given is guided by the maximum tolerated amount of the limiting amino acid (e.g., Phe in PKU). In MMA/PA and UCDs, protein tolerance is used to calculate the daily breast milk’s safe volume (1). Therefore, healthcare professionals must carefully calculate how much IPS and breast milk infants receive, based on the nutritional content of the IPSs. In Italy, a variety of products are available to meet the nutritional needs of neonatal and infant patients with IMDs. However, there are differences in the nutritional composition of these IPSs compared to those intended for healthy children. A detailed comparison of macronutrients, micronutrients, and functional components is essential to evaluate their content and improve dietary management in IMD patients.

### 1.1 European regulation for protein substitutes for infants with IMDs

The nutritional composition of infant and follow-on formulas available in the European Union (EU) must meet the nutritional requirements of infants as stated in the Commission Delegated Regulation (EU) 2016/127 of 25 September 2015 (CDR-EU 2016/127) which supplements the Regulation (EU) No 609/2013 of the European Parliament and of the Council of 12 June 2013 (4), defining the specific requirements for composition and information requirements for infants and follow-on formulas. PSs for infants with IMDs in the EU are classified as Foods for Special Medical Purposes (FMSPs). These PSs must meet the nutritional requirements outlined in the Commission Delegated Regulation (EU) 2016/128 of 25 September 2015 (CDR-EU 2016/128), which supplements Regulation (EU) No 609/2013 of the European Parliament and the Council (5), regarding the specific composition and information requirements for FMSPs. The CDR-EU 2016/128 states that FMSPs, developed to meet the nutritional needs of infants with IMDs, must comply with CDR-EU 2016/127, except for vitamins and minerals, whose contents are specifically defined by the CDR-EU 2016/128. The specific composition requirements for PSs for infants with IMDs are reported in Supplementary Table 1.

### 1.2 Inherited metabolic diseases requiring protein substitutes

Among IMDs, aminoacidopathies, organic acidemias, and urea cycle disorders require special diets with controlled intake of protein and/or AAs. Special IPSs, available from birth, address the nutritional requirements of infants with PKU, tyrosinemia, MSUD, GA1, HCU, IVA, MMA, PA, and UCDs. PKU has been addressed in a recently published paper (6) and for this reason, will not be included in this review. The clinical description of IMDs included in this review is reported in Supplementary material 1, while Table 1 summarizes the main characteristics of tyrosinemia type 1, MSUD, GA1, HCU, IVA, MMA, PA, and UCDs.

**Table 1:** A summary table of the main etiological and dietetic characteristics of each IMD is included in this review.

IMD	Main etiological characteristics and dietary management
Tyrosinemia type 1	It is caused by an enzyme defect of fumarylacetoacetate hydrolase (FAH), leading to the accumulation of tyrosine and its toxic metabolites, such as succinyl acetone (SA). A low Phenylalanine (Phe) and Tyrosine (Tyr) diet is of utmost importance in reducing the risk of complications.
MSUD	MSUD is caused by homozygous or compound heterozygous mutations in one or more subunits of the mitochondrial branched-chain α-ketoacid dehydrogenase (BCKAD) complex. Patients must follow a low-natural protein diet to regulate and minimize the intake of branched-chain amino acids (BCAAs) while ensuring adequate levels of protein, fluid, and energy for optimal growth and development. The tolerable levels of BCAAs depend on age, weight, and BCAAs concentration in the blood. PSs free of BCAAs are required according to tolerance.
GA1	GA1 is caused by autosomal recessive mutations in the GCDH gene, encoding glutaryl-CoA dehydrogenase, a mitochondrial enzyme that plays a role in the degradation of glutaryl-CoA to crotonyl-CoA. Dietary treatment should consist of a low-lysine diet, as this AA is the precursor of glutaric acid and 3-hydroxyglutaric acid synthesis.
HCU	HCU is caused by the deficiency of cystathionine β-synthase, resulting in an abnormal accumulation of homocysteine and its metabolites in the blood and urine. Individuals who do not respond to vitamin B6 require a methionine-restricted diet through a low natural protein intake and PSs free of methionine.
MMA, PA, and IVA	MMA is caused by a deficiency of methyl malonyl-CoA mutase, a vitamin B12-dependent mitochondrial enzyme that catalyzes the conversion of methyl malonyl-CoA to succinyl-CoA; PA is caused by a deficiency of propionyl-CoA carboxylase, a mitochondrial biotin-dependent enzyme which converts propionyl-CoA to methyl malonyl-CoA; IVA is due to a deficiency of isovaleryl-CoA dehydrogenase, on the leucine catabolic pathway. The mainstay of the long-term treatment is a low natural protein and high-energy diet, with specific PSs, free/low of the toxic amino acid precursors (Threonine, Methionine, Valine, and Isoleucine for MMA/PA, Leucine for IVA).
UCDs	UCDs are inborn errors of nitrogen detoxification/arginine synthesis due to defects in the urea cycle enzymes. Treatment depends on the specific enzymatic defect and includes a combination of pharmacological agents, a low natural protein diet, and appropriate nutritional supplementations (i.e., EAAs or BCAAs), to reduce hyperammonemia and/or improve metabolic stability.

## 2 Aims and methods

This study aims to analyze the differences in macronutrients, micronutrients, and other functional components among IPSs currently available on the market in Italy for the dietary management of the following IMDs: tyrosinemia, MSUD, GA1, HCU, IVA, MMA, PA, and UCD. These IMDs are currently identified through the expanded newborn screening program in Italy. In this review, semi-solid PSs specifically designed for complementary feeding were excluded. From March to June 2024, researchers contacted all companies producing and/or marketing IPSs for these specific IMDs in Italy. They requested updated, detailed nutritional information on products suitable for patients up to 1 year of age. A total of 11 powdered IPSs were included in this review. When expressed per 100 g, the nutritional composition of each PS was converted to 100 mL of liquid, according to the dilution recommended by companies. A second analysis allowed us to compare the nutritional values expressed per 100 kcal of product with those reported in the CDR-EU 2016/127. For micronutrient content (vitamins and minerals), comparisons were made with the standards set in the CDR-EU 2016/128. A PS was considered adequate when its nutrient composition complied with the ranges specified in the CDR-EU 2016/127 and 2016/128 regulations. For any values falling outside the regulatory ranges, the percentage deviation was calculated relative to the lower limit (if below range) or the upper limit (if above range).

## 3 Results

### 3.1 Tyrosinemia type 1

Two powdered PSs for infants with tyrosinemia were included in this review. The nutritional composition for 100 mL of PS is reported in Supplementary Table 2a, while Supplementary Table 2b outlines the food components present in 100 kcal of IPSs compared to the CDR-EU 2016/127 of 25 September 2015, apart from vitamins and minerals that have been compared to the CDR-EU 2016/128 of 25 September 2015.

For all IMDs included in this review, Table 2 compares energy, fats (total, saturated, monounsaturated, and polyunsaturated fats; linoleic, alpha-linoleic, docosahexaenoic, arachidonic and eicosapentaenoic acids) carbohydrates [total, sugars, lactose, fiber, galacto-oligosaccharides (GOS) and fructo-oligosaccharides (FOS)], protein equivalents, and vitamins or minerals only when not complying with legislation.

**Table 2:** Main food components (energy, main macronutrients, and main vitamins and minerals) contained per 100 kcal of infant protein substitute (IPS) compared to the commission delegated regulation (EU) 2016/127 of 25 September 2015 and to the commission delegated regulation (EU) 2016/128 of 25 September 2015 for vitamins and minerals. Project funded under the National Recovery and Resilience Plan (NRRP), Mission 4 Component 2 Investment 1.3-Call for tender No. 341 of 15 March 2022 of Italian Ministry of University and Research funded by the European Union–NextGenerationEU; Project code PE00000003, Concession Decree No. 1550 of 11 October 2022 adopted by the Italian Ministry of University and Research, CUP D93C22000890001, Project title “ON Foods-Research and innovation network on food and nutrition Sustainability, Safety and Security–Working ON Foods”.

Content per 100 mL	Unit	IPS 1.1	IPS 1.2	IPS 2.1	IPS 2.2	IPS 3.1	IPS 3.2	IPS 4.1	IPS 4.2	IPS 5.1	IPS 5.2	IPS 5.3	MIN EU 2016/127	MAX EU 2016/127
From birth														
Energy	Kj	**318! (+9%)**	293	**318! (+9%)**	293	**319! (+9%)**	293	**320! (+9%)**	293	293	**319! (+9%)**	293	250	293
Energy	Kcal	**76! (+9%)**	70	**76! (+9%)**	70	**76! (+9%)**	70	**76! (+9%)**	70	70	**76! (+9%)**	70	60	70

Content per 100 kcal	Unit	IPS 1.1	IPS 1.2	IPS 2.1	IPS 2.2	IPS 3.1	IPS 3.2	IPS 4.1	IPS 4.2	IPS 5.1	IPS 5.2	IPS 5.3	MIN EU 2016/127	MAX EU 2016/127
Total fats	g	5.26	4.9	5.26	5	5.26	5	5.26	5	5	5.26	5	4.4	6.0
Saturated fatty acids	g	1.05	1.71	1.05	1.71	1.05	1.71	1.05	1.71	1.71	1.05	1.71		
Monounsaturated fatty acids	g	2.11	2.43	2.11	2.43	2.11	2.43	2.11	2.43	2.43	2.11	2.43		
Polyunsaturated fatty acids	g	2.24	0.86	2.24	0.86	2.24	0.86	2.24	0.86	0.86	2.24	0.86		
Linoleic acid	g	**1.86! (+55%)**	0.69	**1.86! (+55%)**	0.69	**1.86! (+55%)**	0.69	**1.86! (+55%)**	0.69	0.69	**1.86! (+55%)**	0.69	0.5	1.2
Alpha-linolenic acid	g	**0.39! (+290%)**	0.07	**0.39! (+290%)**	0.07	**0.39! (+290%)**	0.07	**0.39! (+290%)**	0.07	0.07	**0.39! (+290%)**	0.07	0.05	0.1
Docosahexaenoic acid (DHA)	g (mg)	0.02 (22.37)	0.03 (25.5)	0.02 (22.37)	0.03 (25.5)	0.02 (22.37)	0.03 (25.5)	0.02 (22.37)	0.03 (25.5)	0.03 (25.5)	0.02 (22.37)	0.03 (25.5)	0.02 (20)	0.05 (50)
Arachidonic acid (ARA)	g (mg)	0	0.03 (25.5)	0	0.03 (25.5)	0	0.03 (25.5)	0	0.03 (25.5)	0.03 (25.5)	0	0.03 (25.5)		
Eicosapentaenoic acid (EPA)	mg	4	0.07	4	0.07	4	0.07	4	0.07	0.07	4	0.07	#	

Content per 100 kcal	**Unit**	**IPS 1.1**	**IPS 1.2**	**IPS 2.1**	**IPS 2.2**	**IPS 3.1**	**IPS 3.2**	**IPS 4.1**	**IPS 4.2**	**IPS 5.1**	**IPS 5.2**	**IPS 5.3**	**MIN** **EU 2016/127**	**MAX** **EU 2016/127**
Carbohydrates	g	10.53	10.8	10.53	10.8	10.53	10.8	10.53	10.8	10.71	10.53	10.71	9	14
Sugars	g	5.26	1.57	4.74	1.57	5.26	1.57	5.26	1.57	1.57	4.61	1.57		
Lactose	g	4.61	**0.36! (−92%)**	4.61	**0.36! (−92%)**	4.61	**0.36! (−92%)**	4.61	**0.36! (−92%)**	**0.36! (−92%)**	4.61	**0.36! (−92%)**	4.5￡	**/**
Dietary fibre	g	0	0.8	0	0.8	0	0.8	0	0.8	0.8	0	0.8		

Content per 100 mL	**Unit**	**IPS 1.1**	**IPS 1.2**	**IPS 2.1**	**IPS 2.2**	**IPS 3.1**	**IPS 3.2**	**IPS 4.1**	**IPS 4.2**	**IPS 5.1**	**IPS 5.2**	**IPS 5.3**		
GOS	g	ND	0.48	ND	0.48	ND	0.48	ND	0.48	0.48	ND	0.48	**^**	
FOS	g	ND	0.08	ND	0.08	ND	0.08	ND	0.08	0.08	ND	0.08	**^**	

Content per 100 kcal	**Unit**	**IPS 1.1**	**IPS 1.2**	**IPS 2.1**	**IPS 2.2**	**IPS 3.1**	**IPS 3.2**	**IPS 4.1**	**IPS 4.2**	**IPS 5.1**	**IPS 5.2**	**IPS 5.3**	**MIN** **EU 2016/128**	**MAX** **EU 2016/128**
Protein equivalents	g	2.24	2.86	2.11	2.86	2.24	2.86	2.24	2.86	2.86	2.24	2.86		

Content per 100 kcal	**Unit**	**IPS 1.1**	**IPS 1.2**	**IPS 2.1**	**IPS 2.2**	**IPS 3.1**	**IPS 3.2**	**IPS 4.1**	**IPS 4.2**	**IPS 5.1**	**IPS 5.2**	**IPS 5.3**	**MIN** **EU 2016/127**	**MAX** **EU 2016/127**
Vitamin D3	μg	**1.32! (−34%)**	2.4	**1.32! (−34%)**	2.4	**1.32! (−34%)**	2.4	**1.32! (−34%)**	2.4	2.4	**1.32! (−34%)**	2.4	2	3
Choline	mg	**15.79! (−37%)**	31.29	**15.79! (−37%)**	31.29	**15.79! (−37%)**	31.29	**15.79! (−37%)**	31.29	31.29	**15.79! (−37%)**	31.29	25	50
Iodine	μg	**9.21! (−39%)**	21	**9.21! (−39%)**	21	**9.21! (−39%)**	21	**9.21! (−39%)**	21	21	**9.21! (−39%)**	21	15	35
Selenium	μg	**2.63! (−12%)**	3.8	**2.63! (−12%)**	3.8	**2.63! (−12%)**	3.8	**2.63! (−12%)**	3.8	3.8	**2.63! (−12%)**	3.8	3	8.6

#It is recommended that levels of EPA should not exceed those of DHA. ￡Except for formulas mainly based on soy proteins and those specifically created without lactose for dedicated uses. ^ FOS and GOS may be added to infant formula, in which case their content shall not exceed 0.8 g/100 mL in a combination of 90% oligogalactosyl-lactose and 10% high-molecular-weight oligofructosyl-saccharose (Commission Delegated Regulation (EU) 2016/127 of 25 September 2015). Explanation of IPSs: IPS 1.1: MamoXi Zero TP Infant; IPS 1.2: Nutricia TYR Anamix Infant; IPS 2.1: MamoXi Zero Vil Infant Mix; IPS 2.2: Nutricia MSUD Anamix Infant; IPS 3.1: MamoXi Zero LYS Infant Mix; IPS 3.2: Nutricia GA1 Anamix Infant; IPS 4.1: MamoXi Zero Met Infant Mix^LCP^; IPS 4.2: Nutricia Anamix Infant HCU; IPS 5.1: Nutricia IVA Anamix Infant; IPS 5.2: MamoXi Zero TVMi Infant Mix^LCP^; IPS 5.3 Nutricia MMA/PA Anamix Infant.

A comparative analysis of the energy content of the two IPSs available reveals a range of 70 to 76 kcal/100 mL, which exceeds the maximum value of 70 kcal/100 mL specified by the CDR-EU 2016/127. The protein equivalent (P.Eq.) content of the two products appears to be similar, with both offering a complete amino acid profile, except for L-phenylalanine and L-tyrosine, which are toxic in cases of tyrosinemia. Regarding carbohydrates, the content of the two PSs falls within the specified range of 9–14 g/100 kcal, as outlined in the CDR-EU 2016/127 of 25 September 2015. IPS 1.2 contains a minimal quantity of fiber. IPS 1.1 contains lactose, whereas the lactose content of IPS 1.2 is below the regulatory limit. Only IPS 1.2 contains GOS and FOS, complying with the maximum limit of 0.8 g/100 mL (CDR-EU 2016/127), but do not exactly meet the GOS/FOS ratio of 9:1. The IPS content of fatty acids is within the specified range of 4.4–6 g/100 kcal, as outlined in the CDR-EU 2016/127. DHA is present in both PSs, ranging from 20 to 50 mg/100 kcal. Considering the micronutrient content and its alignment with the requirements outlined in the CDR-EU 2016/127 of 25 September 2015, the iron and calcium content meets the criteria outlined in the Regulation. Zinc and selenium are present in both PSs; however, IPS 1.1 does not meet the selenium requirement, containing 2.58 mg/100 kcal, below the minimum of 3 mg/100 kcal. Concerning the vitamin D content of the products in question, PS 1.1 fails to comply with the specified range of 2–3 μg/100 kcal, as outlined in the CDR-EU 2016/127. For vitamin B12, both IPSs were found to contain this vitamin following the Regulation. Choline is present in both IPSs, but in IPS 1.1 the minimum amount specified in the CDR-EU 2016/127 is not observed. Inositol is present in both formulas and falls within the values established by the CDR-EU 2016/127. Specifically, in IPS 1.1 it is present as myo-inositol.

### 3.2 Leucinosis or maple syrup urine disease

We compared two powdered IPSs designed for infants with MSUD. The nutritional composition per 100 mL of each product is reported in Supplementary Table 3a, while Supplementary Table 3b compares the nutritional values per 100 kcal with the CDR-EU 2016/127, and, regarding vitamins and minerals, with the CDR-EU 2016/128.

A comparison of the two PSs under study reveals that, in terms of energy content, PS 2.1 has a value of 76 kcal/100 mL, slightly above the maximum limit set by the CDR-EU 2016/127, while PS 2.2 falls within the range. The quantity of protein equivalents (P.Eq.) is relatively similar between the two PSs. Regarding the amino acid composition, both products are devoid of BCAAs (leucine, isoleucine, and valine), as required for the treatment of MSUD. The other amino acids are present in comparable concentrations in both PSs. Concerning carbohydrates, the content of the two PSs is within the specified range of 9–14 g/100 kcal. IPS 2.1 contains lactose, whereas IPS 2.2 contains lactose below the regulatory limit. Only IPS 2.2 contains GOS and FOS, complying with the maximum limit of 0.8 g/100 mL (CDR-EU 2016/127), but do not exactly meet the GOS/FOS ratio of 9:1. In both IPSs, the total fat content falls within the range set by the CDR-EU 2016/127, between 4.4 and 6.0 mg/100 kcal. Both IPSs contain DHA in quantities within the established range. Considering vitamins, the vitamin D content of IPS 1.1 fails to comply with the specified range of 2–3 μg/100 kcal, as outlined in the CDR-EU 2016/127. For vitamin B12, both IPSs were found to contain this vitamin following the Regulation. A comparison of the minerals with the specific requirements set out in the CDR-EU 2016/128 reveals that iodine and selenium are present in both IPSs; however, IPS 2.1 does not meet the requirements, containing 2.58 μg/100 kcal of selenium (falling below the minimum of 3 μg/100 kcal) and 9.11 μg/100 kcal of iodine (falling below the minimum of 15 μg/100 Kcal). Choline is present in both IPSs, but in IPS 2.1 the minimum amount specified in the CDR-EU 2016/127 is not observed. Inositol is present in both formulas and falls within the values established by the CDR-EU 2016/127. Specifically, in IPS 2.1 it is present as myo-inositol. The remaining micronutrients fall within the established ranges.

### 3.3 Glutaric aciduria type 1

We compared the nutritional composition of two powdered PSs for infants with GA1 (Supplementary Table 4). Supplementary Table 4b shows the comparison between the nutritional composition of the two selected products and the nutritional values per 100 kcal established by the CDR-EU 2016/127, while the vitamin and mineral content was compared with the values indicated by the CDR-EU 2016/128.

In terms of energy content, the CDR-EU 2016/127 establishes a range of 60–70 kcal/100 mL, which is respected by the IPS 3.2 and slightly exceeded by the IPS 3.1, with a declared energy content of 76 kcal/100 mL. The amount of protein equivalents (P.Eq.) is quite comparable between the two IPSs and meets the thresholds of the CDR-EU 2016/127, even though a precise comparison cannot be made, given that the Regulation refers to protein content, whereas the IPSs are amino acid blends. Regarding amino acid composition, the GA1 treatment requires the administration of lysine-free, tryptophan-reduced, and arginine-enriched mixtures, and these characteristics are observed in both IPSs. The European regulation specifies only two cut-off values for amino acid composition: L-carnitine should be present at a minimum of 1.2 mg/100 kcal and taurine at a maximum of 12 mg/100 kcal. Both IPSs meet these established limits. The amount of carbohydrates is similar between the two IPSs, fluctuating between the ranges delineated by CDR-EU 2016/127 of 9–14 mg/100 kcal. The presence of fiber is insignificant, amounting to only negligible levels in IPS 3.2. The amount of lactose in IPS 3.1 follows the minimum requirement of 4.5 g/100 kcal as stated in CDR-EU 2016/127. Conversely, IPS 3.2 contains negligible amounts of lactose, at 0.36 g/100 kcal. Only IPS 3.2 contains GOS and FOS, complying with the maximum limit of 0.8 g/100 mL (CDR-EU 2016/127), but do not exactly meet the GOS/FOS ratio of 9:1. Regarding fats, both IPSs contain comparable quantities fully within the ranges established by the current regulatory standards. Neither of the IPSs provides a comprehensive list of the fatty acids present. The amount of saturated fatty acids is comparable between the two IPSs, while the amount of unsaturated fatty acids is not comparable. IPS 3.1 contains 2.38 mg/100 kcal of polyunsaturated fatty acids, whereas IPS 3.2 contains only 0.86 mg/100 kcal. Both IPSs contain amounts of DHA that follow the minimum threshold outlined by CDR-EU 2016/127 (20–50 mg/100 kcal). Considering micronutrients, iron is present in comparable amounts in both IPSs, specifically 1.19 mg in IPS 3.1 versus 1.7 mg/100 kcal in IPS 3.2, which is within the limits set by the CDR-EU 2016/128. The same is true for the other micronutrients, except for iodine and selenium, whose content is below the minimum limit established for the IPS 3.1. Regarding vitamins, the quantities present in the two IPSs exhibit considerable heterogeneity. The vitamin D content is inadequate in IPS 3.1 when compared to the range of 2–3 μg as specified by the regulation. Regarding choline, it is present in both IPSs, but in IPS 3.1 the minimum amount specified in the CDR-EU 2016/127 of 25 September 2015 is not reached. Inositol is present in both formulas and falls within the values established by the CDR-EU 2016/127. Specifically, in IPS 3.1 it is present as myo-inositol.

### 3.4 Classical homocystinuria

This review compares two powdered IPSs for infants with HCU. Supplementary Table 5a presents the nutritional composition per 100 mL of PS, while Supplementary Table 5b compares the food components per 100 kcal of IPS to the requirements outlined in the CDR-EU 2016/127, except for vitamins and minerals, which have been compared to CDR-EU 2016/128.

The energy content of the two IPSs for HCU ranges from 70 kcal to 76 kcal/100 mL, which, in the case of IPS 4.1, exceeds the range of 60–70 kcal/100 mL specified in the CDR-EU 2016/127. The protein equivalent (P.Eq.) content is comparable between the two IPSs, and both products present a complete amino acid profile, except for methionine, which is toxic in individuals with HCU. Regarding carbohydrates, the two IPSs for HCU exhibit comparable levels and both fall within the target range of 9–14 g/100 kcal established by CDR-EU 2016/217. Only IPS 4.2 contains a modest quantity of dietary fiber. The lactose content in IPS 4.1 meets the minimum requirement of 4.5 g/100 kcal, as reported in the CDR-EU 2016/127. On the contrary, IPS 4.2 contains negligible amounts of lactose (0.36 g/100 kcal). Only IPS 4.2 contains GOS and FOS, complying with the maximum limit of 0.8 g/100 mL (CDR-EU 2016/127), but do not exactly meet the GOS/FOS ratio of 9:1. The content of total fat content is quite similar between IPSs, with both formulas falling within the limits defined by the Regulation. The DHA content of the two IPSs is equivalent. A comparison of the micronutrients in both formulas with the specific requirements outlined in the CDR-EU 2016/128 reveals that the iron content in both meets the requirements of this Regulation. Furthermore, the calcium content exhibits a variation within the reference range of 50–250 mg/100 kcal. Additionally, both products contain zinc and selenium, however, for IPS 4.1 the selenium level is below the minimum requirement for an infant protein formula. Regarding vitamin D, the content of IPS 4.1 is below the specified range of 2–3 μg/100 kcal, as outlined in the Regulation. Regarding vitamin B12, the levels observed in both IPSs are comparable and fall within the range specified in the CDR-EU 2016/128. The choline content in IPS 4.1 is below the established minimum value of 25 mg/100 Kcal. Inositol is present in both formulas and falls within the values established by the CDR-EU 2016/127. Specifically, in IPS 4.1 it is present as myo-inositol.

### 3.5 Classic organic acidurias: isovaleric aciduria, propionic aciduria and methylmalonic aciduria

Supplementary Table 6a reports the nutritional comparison of three powdered IPSs for classic organic acidurias. Food components per 100 kcal of IPSs compared to the CDR-EU 2016/127, and for vitamins and minerals to the CDR-EU 2016/128, are reported in Supplementary Table 6b.

Regarding energy content, the range observed is from 70 kcal to 76 kcal/100 mL, which for the IPS 5.2 exceeds the target range of 60–70 kcal/100 mL as outlined in the CDR-EU 2016/127. The three IPSs differ in their protein equivalent content, with values ranging from 2.18 to 2.86 g/100 kcal. However, they all have a complete amino acid profile: IPS 5.1 does not contain leucine, an amino acid toxic for IVA, while IPSs 5.2 and 5.3 do not contain threonine, valine, methionine, and isoleucine, which are toxic for MMA and PA. Concerning carbohydrates, the content is comparable across the three IPSs (10.7 g/100 kcal), and all meet the target range of 9–14 g/100 kcal as specified by the CDR-EU 2016/127. The soluble carbohydrate content of the three IPSs ranged from 1.57 to 4.95 g/100 kcal. The lactose content in IPS 5.2 meets the minimum requirement of 4.5 g/100 kcal, as reported in CDR-EU 2016/127. Conversely, IPSs 5.1 and 5.3 contain negligible amounts of lactose (0.36 g/100 kcal). Only IPS 5.1 and 5.3 contain GOS and FOS, complying with the maximum limit of 0.8 g/100 mL (CDR-EU 2016/127), but do not exactly meet the GOS/FOS ratio of 9:1. A comparison of the total fat and fatty acid content reveals a range of 5 to 5.54 g/100 kcal, which follows the values of 4.4–6 mg/100 kcal established by the CDR-EU 2016/127. A comparative analysis of the micronutrient content of the three IPSs revealed that the iron and calcium levels range from 1.19 to 1.7 mg/100 kcal and from 87.86 to 99 mg/100 kcal, respectively. However, these values were found to satisfy the requirements outlined in the CDR-EU 2016/128. All products contain iodine and selenium, with values falling within the specified range for IPS 5.1 and 5.3, but not for IPS 5.2, with levels of iodine and selenium below the range. Vitamins represent another category of essential micronutrients: the content of vitamin A, vitamin E, vitamin C, and vitamin B12 is comparable across products and within the required range for IPSs. The choline content in IPS 5.2 is below the established minimum value of 25 mg/100 Kcal. Inositol is present in all three formulas and falls within the values established by the CDR-EU 2016/127. Specifically, in IPS 5.2, it is present as myo-inositol.

### 3.6 Urea cycle disorders

There are currently no complete IPSs specifically formulated for infants with UCDs. Instead, the only product that is currently available provides only essential amino acids (EAAs), to be used only in specific cases. Consequently, this formulation cannot be directly compared to the requirements outlined in the CDR-EU 2016/127 for infant formulas, or the vitamin and mineral standards in the CDR-EU 2016/128 for foods for special medical purposes. Supplementary Table 7 provides an overview of the nutritional composition of IPS 6.1. The product is devoid of complete proteins and fats, consisting of a blend of essential amino acids, vitamins, minerals, and trace elements.

## 4 Discussion

The nutritional management of infants with IMDs presents unique challenges due to the specific dietary restrictions required for each disease. PSs designed for infants with IMDs aim to meet their nutritional needs, ensuring the presence of EAAs necessary for growth and development while limiting or eliminating toxic metabolites associated with the condition (7). This is the first review to analyze and highlight the heterogeneity in the nutritional composition of PSs available in Italy for infants with IMDs, specifically tyrosinemia, MSUD, GA1, HCU, IVA, MMA, PA, and UCDs. Notably, PKU has been excluded from this analysis, as it has been the subject of a recent comprehensive review (6), which included 7 IPSs, six powder and one liquid, revealing heterogeneity for the content of EPA, FOS, GOS, human milk oligosaccharides, and nucleotides.

The results show that, for the majority of the IMDs considered (tyrosinemia, MSUD, GA1, HCU, MMA, PA), only two IPSs from two different companies are available. For IVA, only one product is available, while for UCDs, essential amino acids are available from only one company. Regarding energy content, several PSs exceed by 9% the upper limits set by the CDR-EU 2016/127, which established a range of 60–70 kcal/100 mL for infant formulas. Although higher caloric intakes may be necessary in some cases to compensate for metabolic decompensation (8), these discrepancies may also put patients at risk for overfeeding and excessive weight gain, requiring careful clinical evaluation (9). In terms of P.Eq., the analyzed PSs provide a complete amino acid profile except for the AAs toxic to specific IMDs (e.g., phenylalanine and tyrosine for tyrosinemia). However, the concentration of these AAs appears to be similar across products, suggesting general adherence to guidelines for IMD management. The carbohydrate content of IPSs generally follows regulatory requirements, ranging between 9 and 14 g/100 kcal, as outlined in the Delegated Regulation (EU) 2016/127. However, the presence of lactose varies between products. In half of the products, lactose levels are 92% lower than the minimum required by regulations. As human milk’s lactose content is about 7gr/100 mL (10), this sugar should be preferentially employed for infant formulas, providing energy, favoring calcium, magnesium, and zinc absorption and retention, and exerting prebiotic and immunological functions (11). Low lactose content in infant formulas may be associated with alterations of the gut microbiota composition (12). Furthermore, regarding fiber, half of the products do not contain it, while half of them contain only minimal amounts; however, there is no established legislative reference value for fiber content. Regarding this aspect, a notable omission across many of the IPSs is the inclusion of prebiotics such as FOS and GOS, which are common in standard infant formulas (13) and are known to promote gut health and immune function (14). The IPSs containing FOS and GOS respect the maximum limit of 0.8 g/100 mL (CDR-EU 2016/127), but do not exactly meet the GOS/FOS ratio of 9:1, although the difference is small. The Scientific Opinion of the European Food Safety Authority (EFSA) on the essential composition of infant formulas states that it is not currently possible to add a mixture of oligosaccharides that mimics those found in human breast milk (human milk oligosaccharides - HMOs), due to their high variability and structural complexity. They are not considered mandatory components, as current evidence is insufficient to confirm any definitive health benefits for infants from supplementation with GOS and FOS, despite findings that such supplementation increases the abundance of beneficial gut bacteria such as Bifidobacteria and Lactobacilli (15). The increase in Bifidobacteria and Lactobacilli is considered a positive outcome and suggests a functional effect of infant formulas that more closely resembles that of breast milk. However, there is currently no evidence of the long-term impact of infant formula with added prebiotics. Research is ongoing to evaluate the potential benefits of prebiotics in infant nutrition. Regarding fats, most IPSs comply with the regulatory limits of 4.4–6 g/100 kcal as required by European regulations. However, discrepancies exist concerning the presence of essential PUFAs, such as linoleic acid and alpha-linolenic acid, which exceed the maximum value set by the CDR-EU 2016/127 in half of the products, potentially disrupting the omega-6/omega-3 balance. DHA, which is critical for neurological development (16), is included in all analyzed IPSs and is always at the optimal level. Half of the IPSs included do not contain ARA, but all of them contain EPA. According to the CDR-EU 2016/127, EPA content does not exceed DHA content. When ARA is included, its content matches that of DHA. For infant formulas, a position paper underlined the importance of ARA together with DHA (17) while an expert opinion (18) recommended the addition of ARA at concentrations similar to or higher than those of DHA, with a minimum of 0.3% and potentially up to 0.64% of total fatty acids, in line with human milk. ARA, together with DHA, plays a synergistic role in brain and visual development during infancy (19). The imbalance or absence of ARA in these products may disrupt this developmental axis. This is especially relevant for infants with IMDs, whose neurodevelopment may already be vulnerable due to disease-related factors. A detailed micronutrient analysis reveals significant deficiencies in some IPSs, with non-compliance with the recommended levels for several essential minerals. For example, selenium levels in several IPSs are below the minimum established by European Regulation (−12%), potentially increasing the risk of deficiency in neonates, for whom selenium is crucial for antioxidant function (20). Similarly, vitamin D concentrations are often below the recommended 2–3 μg/100 kcal in many IPSs (−34%), which may predispose infants to vitamin D deficiencies with negative effects on bone development (16). Also, choline and iodine are present in insufficient amounts, being, respectively, 37 and 39% lower than the minimum reference value. Iodine is an essential component of thyroid hormones and a particularly critical nutrient for child development. Poor iodine nutrition may impair thyroid hormone synthesis and thereby affect physical, neurological, and intellectual development (21–24). Choline is important for lipid metabolism and brain, liver, and muscle function, playing a crucial role in development and long-term cognitive health (25, 26). Its inadequate inclusion may pose long-term health risks, especially in vulnerable infant populations with compromised metabolic pathways. As for patients with UCDs, they can be breastfed, either expressed or on demand, depending on their metabolic stability and condition, due to the low protein content of human milk (1). An additional protein-free infant formula may be needed in some cases (2). One EAA product is available on the market for UCDs. When a stricter restriction of natural protein intake is necessary, EAAs can be supplemented to help meet the minimum protein requirement, preventing deficiencies and supporting protein synthesis without excessive nitrogen intake (2). The latest evidence shows that a targeted supplementation with BCAAs could be useful, i.e., for patients on sodium phenylbutyrate or phenylacetate/benzoate treatment, to prevent plasma BCAAs deficiency, often reported before metabolic decompensation (27).

Our findings stress both strengths and limitations in the IPSs analyzed, with potential implications for optimizing the dietary management of infants with IMDs. The variations in the nutritional composition among different IPSs available for infants with IMDs in Italy suggest that patients may not be receiving fully optimized dietary management, which could increase the risk of nutritional deficiencies or imbalances. Moreover, the observed variability in macronutrients, micronutrients, and bioactive components raises important questions regarding their potential long-term impact on infant health and development. The low presence of critical micronutrients, such as selenium, iodine, and vitamin D, in conjunction with inconsistency in protein content, could have a detrimental impact on infants’ development. This emphasizes the need for a more complete and balanced nutritional composition of IPSs designed for IMDs, meantime meeting the requirements outlined in the European regulations.

Although breastfeeding and early feeding in PKU have been extensively studied (28), there is a notable lack of literature addressing other IMDs. Additionally, there is a lack of consensus regarding the optimal breastfeeding practices and the use of FMSPs, i.e., amino acid protein substitutes, in IMDs. Breastfeeding, which is feasible in the majority of IMDs affecting protein metabolism (1), should be actively promoted due to its well-documented benefits (29). However, IPSs must be of the highest nutritional quality to meet the specific clinical needs of individuals with IMDs. This lack of uniformity can lead to inconsistencies in patient care and potentially impact the effectiveness of dietary management in IMDs. Therefore, there is a pressing need for comprehensive research that not only broadens the focus beyond PKU but also standardizes infant feeding practices to improve health outcomes for all IMDs. In this context, the systematic review by Ilgaz et al. (30) analyzed data on growth, metabolic and neurodevelopmental status, nutritional and immune profiles, and maternal outcomes related to human milk in the dietary management of IMDs. The review primarily identified studies on PKU, while evidence for other IMDs remains limited. Available data suggest that human milk feeding may be feasible with careful monitoring and disease-specific formula supplementation when necessary. However, no relevant studies were available for many of the assessed outcomes.

## 5 Conclusion and future directions

This review provides the first comprehensive comparative analysis of IPSs available on the Italian market for the dietary management of IMDs, including tyrosinemia type 1, MSUD, GA1, HCU, classic organic acidurias (isovaleric, propionic, and methylmalonic acidurias), and UCDs. The nutritional management of infants with IMDs requires a delicate balance between meeting the dietary restrictions imposed by the metabolic disorder and ensuring adequate nutritional intake to support growth and development. The IPSs available in Italy provide a solid basis for managing IMDs, but exhibit wide variability in their composition, particularly concerning energy density, micronutrients, essential fatty acids, and prebiotics. The results of this review reveal considerable heterogeneity in the nutritional composition of IPSs, with several products exceeding energy recommendations (+9%) and showing significant deviations in key nutrients. Linoleic acid and alpha-linolenic acid were found at levels 55 and 290% above regulatory limits, respectively, while lactose, vitamin D, choline, selenium, and iodine levels were frequently below the minimum recommended values (−92%, −34%, −37%, −12%, and −39%, respectively). Although all IPSs contained DHA and EPA, only half included ARA.

While the CDR-EU (EU) 2016/127 and 2016/128 provide clear guidelines for the nutritional composition of infant formulas, the deviations observed in this review suggest that further regulatory oversight may be needed for IPSs intended for infants with IMDs. This regulatory gap may contribute to inconsistencies in nutritional composition among IPSs, underscoring the need for targeted legislative measures to ensure that the nutritional needs of this particularly vulnerable population are adequately and uniformly met. A more standardized approach may ensure that all IPSs consistently meet the essential nutritional requirements, particularly for vulnerable populations such as infants with IMDs. Looking forward, there is a need for the development of more comprehensive and balanced IPSs that not only meet the specific AAs restrictions, but also provide adequate levels of all essential micronutrients, essential fatty acids, and functional ingredients such as prebiotics. Furthermore, given the advances in nutritional science, future products could benefit from the inclusion of bioactive components that support gut health, immune function, and neurodevelopment.

## Data Availability

The original contributions presented in the study are included in the article/Supplementary material, further inquiries can be directed to the corresponding author/s.
