# Supplementary material for: Are protein substitutes available in Italy for infants with inherited metabolic diseases all the same?
Source: Front Nutr. 2025 May 26;12:1581295. doi: 10.3389/fnut.2025.1581295 (PMC12146189; doi:10.3389/fnut.2025.1581295)
Supplement: Supplementary file 1 [file Table_1.docx]

Supplementary Material

# Supplementary Material 1: A description of IMDs is included in this review.

**1. Tyrosinemia type I**

Tyrosinemia type 1 is a rare IMD with an estimated incidence of 1:120,000 in Europe (1). It is caused by an enzyme defect of fumarylacetoacetate hydrolase (FAH) in the tyrosine degradation pathway. The defect of FAH leads to the accumulation of tyrosine and its toxic metabolites, such as succinylacetone (SA). SA serves as a surrogate parameter of toxicity. The accumulation of toxic metabolites, particularly in the kidneys, liver, and nervous system, results in organ damage and subsequent hepatic failure, which may be accompanied by comorbidities affecting the renal and neurologic systems (1). The clinical symptoms of this condition typically manifest before the age of two years, with many children presenting with evidence of acute liver failure and renal dysfunction within the first six months of age. Neurological crises, manifesting as painful episodes affecting extremity and/or abdominal function, accompanied by hypertension and hyponatremia, may present at any time and may result in respiratory failure and death (2). All children with tyrosinemia type 1 are at high risk for hepatocellular carcinoma, and this may also be the first recognized clinical feature (3). Thus, initiating a comprehensive management strategy is mandatory as soon as the diagnosis is made. An effective medical treatment with 2-[2-nitro-4-trifluoromethylbenzoyl]-1,3-cyclohexanedione (NTBC, nitisinone) exists but requires early identification of affected children for optimal long-term results. Initial efforts at the newborn screening to detect presymptomatic infants with tyrosinemia type 1 by measuring tyrosine levels identified some, but not all, affected children (2), hence a second-tier test assessing succinylacetone concentration on the same dried blood spot has been introduced to improve the newborn screening efficacy. As previously mentioned, nitisinone has dramatically improved the clinical outcome of patients with tyrosinemia type 1. However, dietary treatment remains a fundamental component of the management plan, as nitisinone inhibits tyrosine catabolism at an early metabolic level, causing iatrogenic hypertyrosinemia. Hence, a low phenylalanine and tyrosine diet is paramount to reduce the risk of cutaneous and ocular complications (4,5).

This dietary regimen is comparable to the one used for phenylketonuria, where only phenylalanine intake is restricted, alongside tyrosine supplementation. The primary objective of dietary therapy in tyrosinemia type 1 is to ensure adequate nutrition to support normal growth and development, while strictly controlling tyrosine levels in the blood and tissues. Although no well-defined target levels are available, tyrosine concentrations below 400 μmol/L may be considered safe. To reach this goal, a diet restricted in natural protein and supplemented with a special tyrosine- and phenylalanine-free amino acid mixture is required. During the first 5-6 months of life, the infant’s diet is exclusively based on breast milk or formula, supplemented with a special tyrosine- and phenylalanine-free infant amino acid mixture. The infant can consume the special tyrosine- and phenylalanine-free infant formula *ad libitum*. Throughout the first year of life, it is recommended that dietitians regularly calculate nutrient intake to ensure the supply of energy and natural protein, as well as tyrosine intake, is appropriate for the infant’s weight development and blood tyrosine levels (5). The market offers different PSs containing mixtures of L-amino acids devoid of phenylalanine and tyrosine. These substitutes are supplemented with essential vitamins and minerals, ensuring the diet provides all necessary amino acids and micronutrients for healthy growth and development. Such products are crucial in managing tyrosinemia type 1, as they help maintain safe tyrosine levels while ensuring the patient’s nutritional needs are met.

**2. Leucinosis or maple syrup urine disease (MSUD)**

MSUD is one of the most prevalent aminoacidopathies, with an incidence of approximately 1:185,000 newborns worldwide. It is an autosomal recessive disorder caused by homozygous or compound heterozygous mutations in one or more subunits of the mitochondrial branched-chain α-ketoacid dehydrogenase (BCKAD) complex, resulting in reduced function. BCKAD catalyzes the second step in the catabolic pathway of branched-chain amino acids (BCAAs), starting the oxidative decarboxylation of α-ketoacids (6). This process involves the conversion of α-ketoacids into acetoacetate, acetyl-CoA, and succinyl-CoA. Consequently, in children with MSUD, BCAAs leucine, valine, isoleucine, and alloisoleucine are elevated in the blood, cerebrospinal fluid, urine, and other body fluids. This results in a spectrum of symptoms, including immune system dysfunction, skeletal muscles, and central nervous system (6). The primary effects on the central nervous system are mainly due to the reduction of glutamate and the accumulation of leucine and α-ketoisocaproic acid. Indeed, in the classical neonatal form, the characteristic maple syrup odor of the urine after birth is accompanied by various neurological symptoms during the first week of life. These include irritability, lethargy, feeding difficulties, apnea, opisthotonus, "pedaling" movements, and finally, coma and early death due to cerebral edema (7,8). Conversely, intermediate, intermittent, and thiamine-responsive forms are typically asymptomatic at birth. In these cases, symptoms may manifest at any time in life, with decompensation occurring during illness or stress. Indeed, fever, infections, surgery, trauma, or physical exertion induce the catabolism of endogenous proteins, thereby increasing the risk of acute leucine intoxication and neurological deterioration in children with MSUD (9). Given the incidence of the disease and its favorable response to early dietary treatment, neonatal screening is currently recommended to detect high plasma concentrations of BCAAs early on.

MSUD patients must follow a stringent low-protein diet to regulate and minimize the intake of BCAAs while ensuring adequate levels of protein, fluid, and energy for optimal growth and development. The tolerable levels of BCAAs in children depend on their age, height, weight, and BCAA concentration in the blood. In addition to dietary therapy, a thiamin challenge with a dosage of 50-200 mg/day is considered for four weeks to evaluate thiamine responsiveness. It is recommended that patients maintain a BCAAs-restricted diet during the assessment of the thiamine response, and throughout long-term supplementation (10). Nutrition management includes BCAAs-free medical foods specifically formulated to provide 80–90% of protein requirements, as well as most of the energy and micronutrient necessities throughout life (11). The nutritional requirement for leucine is typically met through breast milk or infant formula. Once an infant has reached the developmental stage at which they can consume solid foods, the leucine present in breast milk or infant formula is replaced by that found in these foods. The leucine daily requirement determines the rate of dietary protein restriction. It is recommended that valine and isoleucine be supplemented, as their content in medical foods is often lower than that of leucine (12).

**3. Glutaric aciduria type 1 (GA1)**

GA1 is a rare metabolic disorder in which the deficient catabolism of the amino acids lysine, hydroxylysine, and tryptophan results in the accumulation of neurotoxic substances. Its worldwide incidence ranges between 1:90,000 and 1:120,000 newborns. It is caused by autosomal recessive mutations in the GCDH gene located on chromosome 19. This gene encodes for glutaryl-CoA dehydrogenase, a mitochondrial enzyme that plays a role in the degradation of glutaryl-CoA to crotonyl-CoA (13). The lack of function of GCDH results in the accumulation of glutaric acid and 3-hydroxyglutaric acid mainly in brain tissue, where they induce oxidative stress, neuroinflammation, dysregulation of neurotransmission, bioenergetic dysfunctions and alterations in vessels and blood-brain barrier, thus causing cerebral injuries (14). At birth, children affected by GA1 may present with unspecific clinical features such as macrocephaly, otherwise being asymptomatic. However, in case of delayed diagnosis, most of them develop neurological impairments after an acute encephalopathic crisis triggered by fever, fasting, diarrhea, or vomiting, occurring in a vulnerable age phase (between 3-36 months of life), with concomitant massive accumulation of glutaric acid and 3-hydroxyglutaric acid in the brain. This irreversible damage affects the striatal body, predominantly the putamen, resulting in complex muscular dystonia (13). In addition to the neurologic involvement, it has been documented that a small number of patients may develop chronic kidney disease, regardless of the severity of muscular dystonia (15). GA1 is among the IMDs included in the expanded newborn screening program in most countries including Italy. GA1 early detection has been demonstrated to reduce morbidity in affected patients, as it permits the initiation of a specific dietary regimen at an early stage. Indeed, once irreversible neurological damage has occurred, an effective treatment is not yet available (16). Adherence to the dietary recommendations has been shown to reduce the incidence of some insidious-onset forms of GA1, in which symptoms of brain impairment appear gradually, involving striatal and extra-striatal manifestations, without a history of acute crisis (13). The third revision of the recommendations for diagnosing and managing individuals with GA1 indicates that dietary treatment should consist of a low-lysine diet (13), as lysine is the main precursor of glutaric acid and 3-hydroxyglutaric acid synthesis. Daily protein intake should be integrated using lysine-free, tryptophan-reduced, and arginine-enriched mixtures (AAM). After 6 years of age, due to overcoming this vulnerable phase, a protein-controlled diet, avoiding lysine-rich aliments may be allowed, with consequent AAM withdrawal. The rationale for arginine supplementation in AAM is that this amino acid competes with lysine to cross the blood-brain barrier and the gastrointestinal wall via a membrane transporter. Consequently, the administration of arginine-rich formulas results in a more pronounced reduction in lysine plasma and cerebral concentrations compared with isolated lysine restriction (17). Since in GA1 carnitine binds with glutaryl-CoA generating glutarylcarnitine, a water-soluble and harmless metabolite, oral L-carnitine supplementation should be administered in addition to AAM to compensate for carnitine depletion and to reduce the oxidative stress (13). Emergency treatment must be started promptly in any catabolic event. It consists of contrasting catabolism by administering high-energy infusions enriched with insulin if hyperglycemia occurs, and by correcting dehydration or electrolyte disturbances; moreover, dietary protein intake should be interrupted for 24-48 hours, and L-carnitine supplementation increased (13). As for breastfeeding, the most recent recommendations indicate that it should be encouraged on demand, following the administration of calculated quantities of lysine-free formulas (13). According to The British Inherited Metabolic Diseases Group (BIMDG) guidelines (18,19) recently developed a spreadsheet for calculating the optimal amount of specific lysine-free formulas to be prescribed, based on the infant’s weight. This calculation estimates the quantity of lysine present in the volume of human milk the infant is likely to consume daily thereby ensuring that the daily lysine intake remains below the safe threshold levels.

**4. Classic homocystinuria (HCU)**

Classic HCU is an autosomal recessive disorder of methionine metabolism caused by the deficiency of cystathionine β-synthase, resulting in an abnormal accumulation of homocysteine and its metabolites in the blood and urine. Homocysteine is toxic to cells and thus must be detoxified or removed from the body (20). This condition occurs worldwide, but its prevalence varies consistently according to ethnicity and the method of ascertainment. The true population frequency remains unknown, with estimates ranging from 1:1800 to 1:900,000, based on the birth incidence of patients identified through newborn screening and/or estimating clinically ascertained patients. The highest incidence has been reported in Qatar (1:1800), where a high rate of consanguinity and a founder effect exist, with a carrier frequency of approximately 2%. The clinical manifestations of HCU may range from individuals who are asymptomatic to those with severe multi-system disease, with a wide range of ages at first presentation (21); four main organs/systems may be affected:

- Eye: *ectopia lentis* and/or severe myopia
- Skeleton: excessive height and length of the limbs (marfanoid’ habitus), osteoporosis, and bone deformities, such as *pectus excavatum or carinatum, genu valgum*, and scoliosis
- Central nervous system: developmental delay/intellectual disability, seizures, psychiatric and behavioral problems, and extrapyramidal signs
- Vascular system: thromboembolism

The current treatment strategies for HCU primarily focus on correcting the biochemical abnormalities caused by HCU, by controlling the plasma and cellular homocysteine levels. Newborns identified with HCU by newborn screening shortly after birth are treated to maintain low serum homocysteine concentrations. One of the primary treatments for patients with HCU is vitamin B6 supplementation. Pyridoxine is a cofactor of the cystathionine β-synthase and is known to play a critical role in regulating the activity of the cystathionine β-synthase. Unfortunately, not all patients respond to vitamin B6 treatment, so treatment is restricted to those who do respond. In most cases, B6 responders also require a protein-restricted diet to control the production of homocysteine. Individuals who do not respond to vitamin B6 require a methionine-restricted diet, which continues lifelong with regular metabolic monitoring. Methionine restriction results in low protein intake, so a methionine-free amino acid mixture is given to meet infants’ protein requirements and to prevent protein malnutrition. Natural or low-protein foods provide the minimum amount of methionine required. Betaine is sometimes administered in combination with folic acid to patients who do not respond to vitamin B6. Betaine functions as a methyl donor, facilitating the alternative re-methylation pathway, which converts toxic homocysteine to methionine. Vitamin B12 and folate facilitate the conversion of homocysteine to methionine by inducing the activity of methionine synthase, thereby reducing serum homocysteine levels. This strategy is employed when the levels of folate and vitamin B12 in the body are insufficient (20). UK guidelines for infants diagnosed by newborn screening recommend that after testing for vitamin B6 responsiveness, the supply of methionine/intact protein (from human milk or infant formula) should be temporarily discontinued, and a methionine-free formula be administered for 2-4 days to reduce elevated plasma homocysteine concentrations. Subsequently, the patient should be returned to human milk or infant formula to meet his/her minimum daily methionine requirement, supplemented with specific methionine-free PS to prevent protein malnutrition (19).

**5. Isovaleric aciduria (IVA), Propionic aciduria (PA) and Methylmalonic aciduria (MMA)**

The term “classical organic acidurias” conventionally defines three inherited autosomal recessive disorders of branched-chain amino acids: IVA, PA, and MMA. IVA is due to a deficiency of isovaleryl-CoA dehydrogenase, on the leucine catabolic pathway; PA is caused by a deficiency of propionyl-CoA carboxylase, a mitochondrial biotin-dependent enzyme that converts propionyl-CoA to methyl malonyl-CoA, while MMA is caused by a deficiency of methyl malonyl-CoA mutase, a vitamin B12 dependent mitochondrial enzyme which catalyzes the conversion of methyl malonyl-CoA to succinyl-CoA (22). The IVA, PA, and MMA incidences are approximately 1:80,000, 1:100,000-1:150,000, and 1:50,000, respectively (23). The clinical presentation of these disorders is highly variable, ranging from severe forms to asymptomatic presentations. In most cases, they have a neonatal onset, but late-onset forms may occur during infancy, presenting with a heterogeneous clinical picture. In the classical neonatal-onset form, neonates present with acute and life-threatening neurological deterioration within a few hours or first days of life. In most cases, the progression of symptoms is as follows: from poor feeding, failure to thrive, recurrent vomiting with ketoacidosis, progressive weight loss, irritability, generalized hypotonia, and abnormal movements, to lethargy, seizures, coma, or death, if untreated (22). Following the neonatal period, multi-organ signs and symptoms may manifest in many ways, depending on the main organ/system affected (23). The later-onset form may present at any age during infancy, manifesting as acute, chronic, or intermittent symptoms that may be exacerbated or even occur for the first time following a trigger event (24). The clinical manifestations of the disease include intermittent ataxia, abnormal behavior, poor feeding, recurrent vomiting, weight loss, and a range of neurological symptoms (encephalopathy, developmental delay), as well as cardiac and kidney manifestations. The disease’s classical triggers are any situation inducing catabolic stress, including infections, fever, gastroenteritis, vaccinations, prolonged fasting periods, medication, prolonged or intense physical exercise, surgery, or psychological stress, as well as protein overload (24).

The laboratory hallmarks of organic acidurias include metabolic acidosis with an elevated anion gap, elevated lactate, elevated urinary ketone bodies, hyperammonemia, leuko-thrombocytopenia, anemia, and hyperuricemia (22,23). Due to the improved survival rates observed in patients with this condition, long-term complications with selective organ impairment are frequent. These include intellectual disability, cognitive impairment, epilepsy, metabolic stroke, movement disorders, optic neuropathy, hearing impairment, chronic kidney disease (mostly in MMA), cardiomyopathy and prolonged QTc interval (mainly in PA), acute, recurrent acute and chronic pancreatitis, pancytopenia (especially neutropenia) and osteoporosis. Acute forms of metabolic decompensation are the most common causes of death. Early diagnosis and timely treatment are crucial for improving survival and reducing morbidity in these patients. Long-term management aims to achieve normal growth and metabolic stability by maintaining a non-catabolic state (25). The mainstay of the long-term treatment is a low-protein and high-energy diet, with specific amino acid mixture supplementation, free of toxic amino acid precursors (22). The permitted amount of natural protein to be taken should be determined individually, considering factors such as age, growth, metabolic stability, and the severity of the condition. While natural protein intake should be limited, it should at least meet the safe intake levels of the precursor essential amino acids, according to the 2007 FAO/WHO/UNU Report (26). Age-appropriate energy requirements should support any restriction. The treatment for MMA/PA aims to reduce the production of propionic acid and organic acids that are toxic to the body and to prevent episodes of catabolism, through the restriction of propionic acid precursor amino acids (isoleucine, valine, threonine, and methionine), and to avoid prolonged fasting to limit Propionyl-CoA production from oxidation of long odd-chain fatty acids (27). The IVA diet aims to reduce the accumulation of isovaleric acid by limiting the intake of leucine and thus protein. Furthermore, for MMA, PA, and IVA, it is crucial to provide appropriate vitamin and mineral supplementation, in case the essential nutrients recommended dietary intake should not be met through natural protein sources and precursor-free PSs (23). Breastfeeding should be maintained as part of the daily natural protein intake (24). In addition to dietary modifications, other recommended medical treatments include L-carnitine (up to 100 mg/kg/day) to prevent free carnitine deficiency, metronidazole (administered at 10 to 20 mg/kg/day in two to three doses) to reduce gut propionate produced by intestinal bacteria, and vitamin B12 in the responsive forms of MMA (22).

**6. Urea Cycle Disorders (UCDs)**

UCDs are inborn errors of nitrogen detoxification/arginine synthesis due to defects in the urea cycle enzymes, carbamoylphosphate synthetase 1 (CPS1), ornithine transcarbamylase (OTC), argininosuccinate synthetase (ASS), argininosuccinate lyase (ASL) and arginase 1 (ARG1), leading to respective deficiencies (abbreviated CPS1D, OTCD, ASSD, ASLD, and ARG1D) (28). They also include N-acetyl glutamate synthase (NAGS) deficiency, which is associated with deficiency of the N-acetyl glutamate (NAG), the essential activator of CPS1 and the mitochondrial ornithine/citrulline antiporter (ORNT1), causing the hyperornithinemia-hyperammonemia-homocitrullinuria (HHH) syndrome (28). The estimated worldwide incidence is 1:35,000-1:69,000 (28,29). UCDs, if not promptly diagnosed either through neonatal screening test or clinically within the first days of life, cause up to 50% of neonatal hyperammonemias (> 500 μmol/L) with a mortality of 25-50%, and/or frequent neurological sequelae in survivors (28). In addition, hyperammonemia crises may occur at any age, caused by different catabolic events, such as infections, vomiting, fever, or excessive protein load. UCDs may occur with acute or chronic neurological symptoms, such as altered level of consciousness, acute encephalopathy, seizures, psychiatric symptoms (hallucinations, paranoia, mania), lethargy, headache, tremor, ataxia, learning disabilities, and cognitive impairment (28).

The primary goal of UCD therapy is to prevent hyperammonemia neurological complications, by maintaining ammonia blood concentrations below 80 µmol/L, with glutamine levels below 1000 µmol/L, and arginine and essential amino acids and branched-chain amino acids within the normal range (30).

The treatment depends on the specific enzymatic defect including a combination of pharmacological agents, a low natural protein diet, and appropriate nutritional supplementations, to reduce ammonia synthesis and/or hyperammonemia once already present. Ammonia-scavenger drugs include sodium benzoate, phenylbutyrate or phenylacetate, glycerol phenylbutyrate, arginine hydrochloride, citrulline, and carglumic acid. Additionally, as a low natural protein diet is essential to reduce the risk of hyperammonemia, essential amino acid-based PSs are often included in the dietary regimen to help patients meet their daily safe protein intake, without compromising growth, and to prevent endogenous catabolism. Indeed, over time, there has been a growing focus on the quality of PSs, leading to the improvement of such products in terms of bio-functional nutritional factors and micronutrient content, absorption kinetics, and palatability. Thus, these products play a crucial role in the nutritional management of UCDs. Finally, in the event of intercurrent illnesses, a specific emergency regimen must also be implemented (28).

**Supplementary Table 1.** Specific Compositional Nutritional Requirements for Infant Protein Substitutes, defined by the Commission Delegated Regulation (EU) 2016/127 (Table A1) and by the Commission Delegated Regulation (EU) 2016/128 (Table A2).

**Table A1.** Nutritional requirements of infant formula reported in the Commission Delegated Regulation (EU) 2016/127.

| Content per 100 kcal | Unit | MIN | MAX |
| --- | --- | --- | --- |
| Energy | kcal | 60 | 70 |
| L-carnitine | mg | 1.2 |  |
| Taurine! | mg |  | 12 |
| Choline | mg | 25 | 50 |
| Lipids | g | 4.4 | 6 |
| Linoleic acid | mg | 500 (0,5) | 1200 (1,2) |
| Alpha-linolenic acid | mg | 50 (0,05) | 100 (0,1) |
| Docosahexaenoic acid (DHA) | mg | 20 | 50 |
| Eicosapentenoic acid (EPA) # | mg |  |  |
| Phospholipids (g)/L | g |  | 2 |
| Inositol | mg | 4 | 40 |
| Carbohydrates | g | 9 | 14 |
| Lactose ￡ | g | 4.5 |  |
| Fructo-oligosaccharides (FOS) $ | g |  |  |
| Galactooligosaccharides (GOS) $ | g |  |  |
| Nucleotides ˆ | mg |  | 5 |

! The amount of taurine that may be added to infant formulas should not exceed 12 mg/100 kcal. # It is recommended that levels of EPA should not exceed those of DHA. ￡ Except for formulas mainly based on soy proteins and those specifically created without lactose for dedicated uses. $ FOS and GOS may be added but their quantity must be less than 0.8 g/100 mL, with a proportion of 90% GOS and 10% FOS. ˆ If added, the total concentration of nucleotides shall not exceed 5 mg/100 kcal.

**Table A2.** Values for vitamins and minerals in FSMPs developed to meet the nutritional requirements of infants reported in the Commission Delegated Regulation (EU) 2016/128.

| Content per 100 kcal | Unit | MIN | MAX |
| --- | --- | --- | --- |
| Vitamin A | mg-RE | 70 | 180 |
| Vitamin D | mg | 2 | 3 |
| Vitamin K | mg | 1 | 25 |
| Vitamin C | mg | 4 | 30 |
| Vitamin B1, Thiamin | mg | 0.04 | 0.3 |
| Vitamin B2, Riboflavin | mg | 0.06 | 0.45 |
| Vitamin B6 | mg | 0.02 | 0.3 |
| Niacin | mg | 0.4 | 3 |
| Total Folate $ | mg | 9 (DFE=15) | 28.56 (DFE=47.6) |
| Vitamin B12 | mg | 0.1 | 0.5 |
| Pantothenic acid | mg | 0.4 | 2 |
| Biotin | mg | 1 | 20 |
| Vitamin E | mg α-TE | 0.6 | 5 |
| Sodium | mg | 25 | 60 |
| Chloride | mg | 60 | 160 |
| Potassium | mg | 80 | 160 |
| Calcium * | mg | 50 | 250 |
| Phosphorus | mg | 25 | 100 |
| Magnesium | mg | 5 | 15 |
| Iron | mg | 0.3 | 2.5 |
| Zinc | mg | 0.5 | 2.4 |
| Cupper | mg | 0.06 | 0.12 |
| Iodine | mg | 15 | 35 |
| Selenium | mg | 3 | 8.6 |
| Manganese | mg | 0.001 | 0.1 |
| Chromium | mg |  | 10 |
| Molybdenum | mg |  | 14 |
| Fluoride | mg |  | 0.2 |

*The calcium: phosphorus molar ratio shall not be less than 1 nor greater than 2. $ Dietary folate equivalent: 1 μg DFE = 1 μg food folate = 0.6 μg folic acid from FSMPs.

**Supplementary Table 2a.** Food components per 100 mL of infant protein substitute for Tyrosinemia type 1. If nutritional data were not present on the nutritional label, the term “Not Declared” (ND) was used.

| **Content per 100 mL** | **Unit** | **Infant Protein Substitute 1.1** | **Infant Protein Substitute 1.2** |
| --- | --- | --- | --- |
| Energy | Kj | 318 | 293 |
| Energy | kcal | 76 | 70 |
| Total fats | g | 4 | 3.5 |
| Saturated fatty acids | g | 0.8 | 1.2 |
| Monounsaturated fatty acids | g | 1.6 | 1.7 |
| Polyunsaturated fatty acids | g | 1.7 | 0.6 |
| Caproic acid | mg | 0 | 3.98 |
| Caprylic acid | mg | 0 | 59.8 |
| Capric acid | mg | 0 | 48.3 |
| Lauric acid | mg | 0 | 369 |
| Myristic acid | mg | 100 | 160 |
| Palmitic acid | mg | 590 | 241 |
| Palmitoleic acid | mg | 100 | 259 |
| Stearic acid | mg | 110 | 158 |
| Oleic acid | mg | 1560 | 1604 |
| Linoleic acid (LA) | g | 1.41 | 0.48 |
| Alpha-linolenic acid (ALA) | g | 0.30 | 0.05 |
| Docosahexaenoic acid (DHA) | g (mg) | 0.02 (17) | 0.02 (17.8) |
| Arachidonic acid (ARA) | g (mg) | 0 | 0.02 (17.8) |
| Eicosapentaenoic acid (EPA) | mg | 3 | 0.05 |
| Arachidic acid | g | 0.07 | ND |
| Behenic acid | g | 0.02 | ND |
| Cholesterol | g | ND | 0 |
| Carbohydrates | g | 8 | 7.5 |
| Soluble carbohydrates | g | 4 | 1.1 |
| Starch | g | 1.2 | 0 |
| Polyols | g | ND | ND |
| Fructose | g | ND | 0 |
| Maltodextrins | g | 3 | ND |
| Lactose | g | 3.5 | 0.25 |
| D-mannose | g | ND | ND |
| Dietary fibre | g | 0 | 0.56 |
| 2’-Fucosyllactose | g | ND | 0 |
| Lacto-N-tetraose | g | ND | 0 |
| GOS | g | ND | 0.48 |
| FOS | g | 1.7 | 0.08 |
| Protein equivalents | g | 0.08 | 2 |
| L-Alanine | g | 0.12 | 0.10 |
| L-Arginine | g | 0.20 | 0.18 |
| L-Aspartic acid | g | 0.05 | 0.15 |
| L-Cystine | g | ND | 0.07 |
| L-Glutamine | g | 0.11 | 0.02 |
| Glycine | g | 0.09 | 0.16 |
| L-Histidine | g | 0.12 | 0.1 |
| L-Isoleucine | g | 0.2 | 0.16 |
| L-Leucine | g | 0.14 | 0.27 |
| L-Lysine | g | 1.7 | 0.19 |
| L-Methionine | g | 0.05 | 0.04 |
| L-Phenylalanine | g | 0 | 0 |
| L-Proline | g | 0.17 | 0.2 |
| L-Serine | g | 0.11 | 0.12 |
| Taurine | mg | 6 | 4.5 |
| L-Threonine | g | 0.09 | 0.13 |
| L-Tryptophan | g | 0.05 | 0.05 |
| L-Tyrosine | g | 0 | 0 |
| L-Valine | g | 0.14 | 0.18 |
| L-Carnitine | mg | 1.6 | 1.52 |
| L-Glutamic acid | mg | 280 | 201 |
| Nucleotides | mg | ND | 0 |
| Vitamin A | µg RE | 86 | 61.2 |
| Vitamin D3 | µg | 1 | 1.68 |
| Vitamin E | mg α-TE | 0.7 | 1.38 |
| Vitamin C | mg | 9 | 7.34 |
| Vitamin K1 | µg | 3.6 | 5.59 |
| Vitamin K2 (MK-7) | µg | ND | ND |
| Vitamin B1, Thiamin | mg | 0.07 | 0.08 |
| Vitamin B2, Riboflavin | mg | 0.08 | 0.08 |
| Vitamin B3, Niacin | mg | 0.9 | 0.35 |
| Vitamin B6 | mg | 0.08 | 0.08 |
| Total folate | µg | ND | 13.8 |
| Folic acid | µg | 8 | 8.25 |
| Vitamin B12 | µg | 0.2 | 0.18 |
| Biotin | µg | 2 | 2.7 |
| Pantothenic acid | mg | 0.6 | 0.42 |
| Choline | mg | 12 | 21.9 |
| Calcium | mg | 75 | 61.5 |
| Chromium | µg | 4 | 2.1 |
| Copper | mg | 0.05 | 0.06 |
| Iodine | µg | 7 | 14.7 |
| Iron | mg | 1 | 1.19 |
| Magnesium | mg | 8 | 8.75 |
| Manganese | mg | 0.06 | 0.004 |
| Molybdenum | µg | 4 | 1.82 |
| Phosphorus | mg | 50 | 45 |
| Selenium | µg | 2 | 2.66 |
| Zinc | mg | 0.8 | 0.84 |
| Potassium | mg | 75 | 75.8 |
| Sodium | mg | 39 | 28.7 |
| Chloride | mg | 57 | 53.3 |
| Inositol | mg | ND | 14.7 |
| Myo-inositol | mg | 8 | ND |
| Lutein | mg | ND | 0 |
| Fluoride | mg | 0.02 | 0 |
| Salt | g | 0.1 | 0.07 |

**Explanation of infant protein substitutes:** Infant protein substitute 1.1: MamoXi Zero TP Infant; Infant protein substitute 1.2: Nutricia TYR Anamix Infant

**Supplementary Table 2b.** Food components per 100 kcal of infant protein substitute for tyrosinemia type 1 compared to the Commission Delegated Regulation (EU) 2016/127 of 25 September 2015 and to the Commission Delegated Regulation (EU) 2016/128 of 25 September 2015 for vitamins and minerals. If nutritional data were not present on the nutritional label, the term “Not Declared” (ND) was used. A “!” is used when the value does not comply with the Regulation.

| **Content per 100 mL** | **Unit** | **Infant Protein Substitute 1.1** | **Infant Protein Substitute 1.2** | **MIN**  **EU 2016/127** | **MAX**  **EU 2016/127** |
| --- | --- | --- | --- | --- | --- |
|  |  | From birth | From birth |  |  |
| Energy | Kj | **318! (+9%)** | 293 | 250 | 293 |
| Energy | Kcal | **76! (+9%)** | 70 | 60 | 70 |
| **Content per 100 kcal** | **Unit** | **Infant protein substitute 1.1** | **Infant protein substitute 1.2** | **MIN**  **EU 2016/127** | **MAX**  **EU 2016/127** |
| Total fats | g | 5.26 | 4.9 | 4.4 | 6.0 |
| Saturated fatty acids | g | 1.05 | 1.71 |  |  |
| Monounsaturated fatty acids | g | 2.11 | 2.43 |  |  |
| Polyunsaturated fatty acids | g | 2.24 | 0.86 |  |  |
| Caproic acid | mg | 0 | 5.70 |  |  |
| Caprylic acid | mg | 0 | 85.5 |  |  |
| Capric acid | mg | 0 | 69.1 |  |  |
| Lauric acid | mg | 0 | 527 |  |  |
| Myristic acid | mg | 10 | 228 |  |  |
| Palmitic acid | mg | 780 | 344 |  |  |
| Palmitoleic acid | mg | 100 | 3.70 |  |  |
| Stearic acid | mg | 140 | 226 |  |  |
| Oleic acid | mg | 2050 | 2295 |  |  |
| Linoleic acid | g | **1.86! (+55%)** | 0.69 | 0.5 | 1.2 |
| Alpha-linolenic acid | g | **0.39! (+290%)** | 0.07 | 0.05 | 0.1 |
| Docosahexaenoic acid (DHA) | g (mg) | 0.02 (22.37) | 0.03 (25.5) | 0.02 (20) | 0.05 (50) |
| Arachidonic acid (ARA) | g (mg) |  | 0.03 (25.5) |  |  |
| Eicosapentaenoic acid (EPA) | mg | 4 | 0.07 |  |  |
| Arachidic acid | g | 0.09 | ND |  |  |
| Behenic acid | g | 0.03 | ND |  |  |
| Cholesterol | g | ND | 0 |  |  |
| **Content per 100 kcal** | **Unit** | **Infant protein substitute 1.1** | **Infant protein substitute 1.2** | **MIN**  **EU 2016/127** | **MAX**  **EU 2016/127** |
| Carbohydrates | g | 10.53 | 10.8 | 9 | 14 |
| Soluble carbohydrates | g | 5.26 | 1.57 |  |  |
| Starch | g | 1.58 | 0 | / | 2 |
| Polyols | g | ND | ND |  |  |
| Fructose | g | ND | 0 |  |  |
| Maltodextrins | g | 3.95 | ND |  |  |
| Lactose | g | 4.61 | **0.36! (-92%)** | 4.5 | / |
| D-mannose | g | ND | ND |  |  |
| Dietary fibre | g | 0 | 0.8 |  |  |
| 2’-Fucosyllactose | g | ND | 0 |  |  |
| Lacto-N-tetraose | g | ND | 0 |  |  |
| GOS | g | ND | 0.69 |  | ^ |
| FOS | g | ND | 0.11 |  | ^ |
| **Content per 100 mL** | **Unit** | **Infant protein substitute 1.1** | **Infant protein substitute 1.2** |  |  |
| GOS | g | ND | 0.48 |  | ^ |
| FOS | g | ND | 0.08 |  | ^ |
| **Content per 100 kcal** | **Unit** | **Infant protein substitute 1.1** | **Infant protein substitute 1.2** | **MIN**  **EU 2016/127** | **MAX**  **EU 2016/127** |
| Protein equivalents | g | 2.24 | 2.86 |  |  |
| **Content per 100 kcal** | **Unit** | **Infant protein substitute 1.1** | **Infant protein substitute 1.2** | **MIN**  **EU 2016/127** | **MAX**  **EU 2016/127** |
| L-Alanine | g | 0.11 | 0.14 |  |  |
| L-Arginine | g | 0.16 | 0.26 |  |  |
| L-Aspartic acid | g | 0.26 | 0.21 |  |  |
| L-Cystine | g | 0.07 | 0.1 |  |  |
| L-Glutamine | g | ND | 0.03 |  |  |
| Glycine | g | 0.14 | 0.23 |  |  |
| L-Histidine | g | 0.12 | 0.15 |  |  |
| L-Isoleucine | g | 0.16 | 0.23 |  |  |
| L-Leucine | g | 0.26 | 0.39 |  |  |
| L-Lysine | g | 0.18 | 0.27 |  |  |
| L-Methionine | g | 0.07 | 0.06 |  |  |
| L-Phenylalanine | g | 0 | 0 |  |  |
| L-Proline | g | 0.22 | 0.28 |  |  |
| L-Serine | g | 0.14 | 0.17 |  |  |
| Taurine | mg | 7.89 | 6.43 |  | 12 |
| L-Threonine | g | 0.12 | 0.19 |  |  |
| L-Tryptophan | g | 0.07 | 0.08 |  |  |
| L-Tyrosine | g | 0 | 0 |  |  |
| L-Valine | g | 0.18 | 0.26 |  |  |
| L-Carnitine | mg | 2.11 | 2.17 | 1.2 |  |
| L-Glutamic acid | g | 0.37 | 0.29 |  |  |
| Nucleotides# | mg | ND | 0 |  | 5 |
| **Content per 100 kcal** | **Unit** | **Infant protein substitute 1.1** | **Infant protein substitute 1.2** | **MIN**  **EU 2016/127** | **MAX**  **EU 2016/127** |
| Vitamin A | µg RE | 113.16 | 87.5 | 70 | 180 |
| Vitamin D3 | µg | **1.32! (-34%)** | 2.4 | 2 | 3 |
| Vitamin E | mg α-TE | 0.92 | 1.98 | 0.6 | 5 |
| Vitamin C | mg | 11.84 | 10.49 | 4 | 30 |
| Vitamin K1 | µg | 4.74 | 7.99 | 1 | 25 |
| Vitamin K2 (MK-7) | µg | ND | ND |  |  |
| Vitamin B1, Thiamin | mg | 0.09 | 0.11 | 0.04 | 0.3 |
| Vitamin B2, Riboflavin | mg | 0.11 | 0.11 | 0.06 | 0.45 |
| Vitamin B3, Niacin | mg | 1.18 | 0.5 | 0.4 | 3 |
| Vitamin B6 | mg | 0.11 | 0.11 | 0.02 | 0.3 |
| Total folate $ | µg | ND | 19.71 | 9 (DFE = 15) | 28.56  (DFE = 47.6) |
| Folic Acid | µg | 10.53 | 11.79 |  |  |
| Vitamin B12 | µg | 0.26 | 0.26 | 0.1 | 0.5 |
| Biotin | µg | 2.63 | 3.9 | 1 | 20 |
| Pantothenic acid | mg | 0.79 | 0.6 | 0.4 | 2 |
| Choline | mg | **15.79! (-37%)** | 31.29 | 25 | 50 |
| Calcium * | mg | 98.68 | 88.07 | 50 | 250 |
| Chromium | µg | 5.26 | 3 | / | 10 |
| Copper | mg | 0.07 | 0.09 | 0.06 | 0.12 |
| Iodine | µg | **9.21! (-39%)** | 21 | 15 | 35 |
| Iron | mg | 1.32 | 1.7 | 0.3 | 2.5 |
| Magnesium | mg | 10.53 | 12.5 | 5 | 15 |
| Manganese | mg | 0.08 | 0.006 | 0.001 | 0.1 |
| Molybdenum | µg | 5.26 | 2.6 | / | 14 |
| Phosphorus | mg | 65.79 | 64.4 | 25 | 100 |
| Selenium | µg | **2.63! (-12%)** | 3.8 | 3 | 8.6 |
| Zinc | mg | 1.05 | 1.2 | 0.5 | 2.4 |
| Potassium | mg | 98.68 | 108 | 80 | 160 |
| Sodium | mg | 51.32 | 41 | 25 | 60 |
| Chloride | mg | 75.00 | 76.2 | 60 | 160 |
| Inositol | mg | ND | 21 | 4 | 40 |
| Myo-inositol | mg | 10.53 | ND |  |  |
| Lutein | mg | ND | 0 |  |  |
| Fluoride | mg | 0.03 | 0 | / | 0.2 |
| Salt | g | 0.12 | 0.1 |  |  |

^ FOS and GOS may be added to infant formula, in which case their content shall not exceed 0.8 g/100 mL, with a proportion of 90% oligogalactosyl-lactose and 10% high-molecular-weight oligofructosyl-saccharose (Commission Delegated Regulation (EU) 2016/127 of 25 September 2015). * The calcium: phosphorus molar ratio shall not be less than 1 nor greater than 2. # If added, the total concentration of nucleotides shall not exceed 5 mg/100 kcal. $ Dietary folate equivalent: 1 μg DFE = 1 μg food folate = 0.6 μg folic acid from food for special medical purposes. **Explanation of infant protein substitutes:** Infant protein substitute 1.1: MamoXi Zero TP Infant; Infant protein substitute 1.2: Nutricia TYR Anamix Infant.

**Supplementary Table 3a.** Food components per 100 mL of infant protein substitute for MSUD. If nutritional data were not present on the nutritional label, the term “Not Declared” (ND) was used.

| **Content per 100 mL** | | **Unit** | **Infant protein substitute 2.1** | **Infant protein substitute 2.2** |
| --- | --- | --- | --- | --- |
| Energy | | Kj | 318 | 293 |
| Energy | | Kcal | 76 | 70 |
| Total fats | | g | 4 | 3.5 |
| Saturated fatty acids | | g | 0.8 | 1.2 |
| Monounsaturated fatty acids | | g | 1.6 | 1.7 |
| Polyunsaturated fatty acids | | g | 1.7 | 0.6 |
| Caproic acid | | mg | 0 | 98 |
| Caprylic acid | | mg | 0 | 59.8 |
| Capric acid | | mg | 0 | 48.3 |
| Lauric acid | | mg | 0 | 369 |
| Myristic acid | | mg | 10 | 160 |
| Palmitic acid | | mg | 590 | 241 |
| Palmitoleic acid | | mg | 10 | 2.59 |
| Stearic acid | | mg | 110 | 158 |
| Oleic acid | | mg | 1560 | 1604 |
| Linoleic acid (LA) | | mg | 1.41 | 481 |
| Alpha-linolenic acid (ALA) | | mg | 0.30 | 47.6 |
| Docosahexaenoic acid (DHA) | | g (mg) | 0.02 (17) | 0.02 (17.8) |
| Arachidonic acid (ARA) | | g (mg) | 0 | 0.02 (17.8) |
| Eicosapentaenoic acid (EPA) | | mg | 3 | 0.05 |
| Arachidic acid | | g | 0.07 | ND |
| Behenic acid | | g | 0.02 | ND |
| Cholesterol | | g | ND | 0 |
| Carbohydrates | | g | 8 | 7.5 |
| Soluble carbohydrates | | g | 3.6 | 1.1 |
|  | Starch | 1.2 | 1.2 | 0 |
| Polyols | | g | ND | ND |
| Fructose | | g | ND | 0 |
| Maltodextrins | | g | 3 | ND |
| Lactose | | g | 3.5 | 0.25 |
| D-mannose | | g | ND | ND |
| Dietary fibre | | g | 0 | 0.56 |
| 2’-Fucosyllactose | | g | ND | 0 |
| Lacto-N-tetraose | | g | ND | 0 |
| GOS | | g | ND | 0.48 |
| FOS | | g | ND | 0.08 |
| Protein equivalents | | g | 1.6 | 2 |
| L-Alanine | | g | 0.10 | 0.12 |
| L-Arginine | | g | 0.14 | 0.21 |
| L-Aspartic acid | | g | 0.21 | 0.18 |
| L-Cystine | | g | 0.07 | 0.08 |
| L-Glutamine | | g | ND | 0.02 |
| Glycine | | g | 0.12 | 0.19 |
| L-Histidine | | g | 0.09 | 0.12 |
| L-Isoleucine | | g | 0 | 0 |
| L-Leucine | | g | 0 | 0 |
| L-Lysine | | g | 0.18 | 0.22 |
| L-Methionine | | g | 0.05 | 0.05 |
| L-Phenylalanine | | g | 0.11 | 0.14 |
| L-Proline | | g | 0.17 | 0.23 |
| L-Serine | | g | 0.11 | 0.14 |
| Taurine | | mg | 6 | 4.5 |
| L-Threonine | | g | 0.13 | 0.16 |
| L-Tryptophan | | g | 0.05 | 0.06 |
| L-Tyrosine | | g | 0.12 | 0.14 |
| L-Valine | | g | 0 | 0 |
| L-Carnitine | | mg | 1.6 | 1.52 |
| L-Glutamic acid | | g | 0.32 | 0.24 |
| Nucleotides | | mg | ND | 0 |
| Vitamin A | | µg RE | 86 | 61.2 |
| Vitamin D3 | | µg | 1 | 1.68 |
| Vitamin E | | mg α-TE | 0.7 | 1.38 |
| Vitamin C | | mg | 9 | 7.34 |
| Vitamin K1 | | µg | 3.6 | 5.59 |
| Vitamin K2 (MK-7) | | µg | ND | ND |
| Vitamin B1, Thiamin | | mg | 0.07 | 0.08 |
| Vitamin B2, Riboflavin | | mg | 0.08 | 0.08 |
| Vitamin B3, Niacin | | mg | 0.9 | 0.35 |
| Vitamin B6 | | mg | 0.08 | 0.08 |
| Total folate | | µg | ND | 13.8 |
| Folic Acid | | µg | 8 | 8.25 |
| Vitamin B12 | | µg | 0.2 | 0.18 |
| Biotin | | µg | 2 | 2.7 |
| Pantothenic acid | | mg | 0.6 | 0.42 |
| Choline | | mg | 12 | 21.9 |
| Calcium | | mg | 75 | 61.5 |
| Chromium | | µg | 4 | 2.1 |
| Copper | | mg | 0.05 | 0.06 |
| Iodine | | µg | 7 | 14.7 |
| Iron | | mg | 1 | 1.19 |
| Magnesium | | mg | 8 | 8.75 |
| Manganese | | mg | 0.06 | 0.004 |
| Molybdenum | | µg | 4 | 1.82 |
| Phosphorus | | mg | 50 | 45 |
| Selenium | | µg | 2 | 2.66 |
| Zinc | | mg | 0.8 | 0.84 |
| Potassium | | mg | 75 | 75.8 |
| Sodium | | mg | 39 | 28.7 |
| Chloride | | mg | 57 | 53.3 |
| Inositol | | mg | ND | 14.7 |
| Myo-inositol | | mg | 8 | ND |
| Lutein | | mg | ND | 0 |
| Fluoride | | mg | 0.02 | 0 |
| Salt | | g | 0.1 | 0.07 |

**Explanation of infant protein substitutes:** Infant protein substitute 2.1: MamoXi Zero VIL Infant Mix^LCP^; Infant protein substitute 2.2: Nutricia MSUD Anamix Infant.

**Supplementary Table 3b.** Food components per 100 kcal of infant protein substitute for MSUD compared to the Commission Delegated Regulation (EU) 2016/127 of 25 September 2015 and to the Commission Delegated Regulation (EU) 2016/128 of 25 September 2015 for vitamins and minerals. If nutritional data were not present on the nutritional label, the term “Not Declared” (ND) was used. A “!” is used when the value does not comply with the Regulation.

| **Content per 100 mL** | **Unit** | **Infant Protein Substitute 2.1** | **Infant Protein Substitute 2.2** | **MIN**  **EU 2016/127** | **MAX**  **EU 2016/127** |
| --- | --- | --- | --- | --- | --- |
|  |  | From birth | From birth |  |  |
| Energy | Kj | **318! (+9%)** | 293 | 250 | 293 |
| Energy | Kcal | **76! (+9%)** | 70 | 60 | 70 |
| **Content per 100 kcal** | **Unit** | **Infant protein substitute 2.1** | **Infant protein substitute 2.2** | **MIN**  **EU 2016/127** | **MAX**  **EU 2016/127** |
| Total fats | g | 5.26 | 5 | 4.4 | 6.0 |
| Saturated fatty acids | g | 1.05 | 1.71 |  |  |
| Monounsaturated fatty acids | g | 2.11 | 2.43 |  |  |
| Polyunsaturated fatty acids | g | 2.24 | 0.86 |  |  |
| Caproic acid | mg | 0 | 5.7 |  |  |
| Caprylic acid | mg | 0 | 85.5 |  |  |
| Capric acid | mg | 0 | 69.1 |  |  |
| Lauric acid | mg | 0 | 527 |  |  |
| Myristic acid | mg | 10 | 228 |  |  |
| Palmitic acid | mg | 780 | 344 |  |  |
| Palmitoleic acid | mg | 10 | 7.31 |  |  |
| Stearic acid | mg | 140 | 226 |  |  |
| Oleic acid | mg | 2050 | 2295 |  |  |
| Linoleic acid | g | **1.86! (+55%)** | 0.69 | 0.5 | 1.2 |
| Alpha-linolenic acid | g | **0.39! (+290%)** | 0.07 | 0.05 | 0.1 |
| Docosahexaenoic acid (DHA) | g (mg) | 0.02 (22.37) | 0.03 (25.5) | 0.02 (20) | 0.05 (50) |
| Arachidonic acid (ARA) | g (mg) | 0 | 0.03 (25.5) |  |  |
| Eicosapentaenoic acid (EPA) | mg | 4 | 0.07 |  |  |
| Arachidic acid | g | 0.09 | ND |  |  |
| Behenic acid | g | 0.03 | ND |  |  |
| Cholesterol | g | ND | 0 |  |  |
| **Content per 100 kcal** | **Unit** | **Infant protein substitute 2.1** | **Infant protein substitute 2.2** | **MIN**  **EU 2016/127** | **MAX**  **EU 2016/127** |
| Carbohydrates | g | 10.53 | 10.8 | 9 | 14 |
| Soluble carbohydrates | g | 4.74 | 1.57 |  |  |
| Starch | g | 1.58 | 0 | / | 2 |
| Polyols | g | ND | ND |  |  |
| Fructose | g | ND | 0 |  |  |
| Maltodextrins | g | 3.95 | ND |  |  |
| Lactose | g | 4.61 | **0.36! (-92%)** | 4.5 | / |
| D-mannose | g | ND | ND |  |  |
| Dietary fibre | g | 0 | 0.8 |  |  |
| 2’-Fucosyllactose | g | ND | 0 |  |  |
| Lacto-N-tetraose | g | ND | 0 |  |  |
| GOS | g | ND | 0.69 |  | ^ |
| FOS | g | ND | 0.11 |  | ^ |
| **Content per 100 mL** | **Unit** | **Infant protein substitute 2.1** | **Infant protein substitute 2.2** |  |  |
| GOS | g | ND | 0.48 |  | ^ |
| FOS | g | ND | 0.08 |  | ^ |
| **Content per 100 kcal** | **Unit** | **Infant protein substitute 2.1** | **Infant protein substitute 2.2** | **MIN**  **EU 2016/127** | **MAX**  **EU 2016/127** |
| Protein equivalents | g | 2.11 | 2.8 |  |  |
| **Content per 100 kcal** | **Unit** | **Infant protein substitute 2.1** | **Infant protein substitute 2.2** | **MIN**  **EU 2016/127** | **MAX**  **EU 2016/127** |
| L-Alanine | g | 0.13 | 0.17 |  |  |
| L-Arginine | g | 0.18 | 0.3 |  |  |
| L-Aspartic acid | g | 0.28 | 0.26 |  |  |
| L-Cystine | g | 0.09 | 0.11 |  |  |
| L-Glutamine | g | ND | 0.03 |  |  |
| Glycine | g | 0.16 | 0.27 |  |  |
| L-Histidine | g | 0.12 | 0.17 |  |  |
| L-Isoleucine | g | 0 | 0 |  |  |
| L-Leucine | g | 0 | 0 |  |  |
| L-Lysine | g | 0.24 | 0.31 |  |  |
| L-Methionine | g | 0.07 | 0.07 |  |  |
| L-Phenylalanine | g | 0.14 | 0.2 |  |  |
| L-Proline | g | 0.22 | 0.33 |  |  |
| L-Serine | g | 0.14 | 0.2 |  |  |
| Taurine | mg | 7.89 | 6.43 |  | 12 |
| L-Threonine | g | 0.17 | 0.23 |  |  |
| L-Tryptophan | g | 0.07 | 0.09 |  |  |
| L-Tyrosine | g | 0.16 | 0.2 |  |  |
| L-Valine | g | 0 | 0 |  |  |
| L-Carnitine | mg | 2.11 | 2.17 | 1.2 |  |
| L-Glutamic acid | g | 0.42 | 0.34 |  |  |
| Nucleotides # | mg | ND | 0 |  | 5 |
| **Content per 100 kcal** | **Unit** | **Infant protein substitute 2.1** | **Infant protein substitute 2.2** | **MIN**  **EU 2016/127** | **MAX**  **EU 2016/127** |
| Vitamin A | µg RE | 113.16 | 87.5 | 70 | 180 |
| Vitamin D3 | µg | **1.32! (-34%)** | 2.4 | 2 | 3 |
| Vitamin E | mg α-TE | 0.92 | 1.98 | 0.6 | 5 |
| Vitamin C | mg | 11.84 | 10.49 | 4 | 30 |
| Vitamin K1 | µg | 4.74 | 7.99 | 1 | 25 |
| Vitamin K2 (MK-7) | µg | ND | ND |  |  |
| Vitamin B1, Thiamin | mg | 0.09 | 0.11 | 0.04 | 0.3 |
| Vitamin B2, Riboflavin | mg | 0.11 | 0.11 | 0.06 | 0.45 |
| Vitamin B3, Niacin | mg | 1.18 | 0.5 | 0.4 | 3 |
| Vitamin B6 | mg | 0.11 | 0.11 | 0.02 | 0.3 |
| Total folate $ | µg | ND | 19.71 | 9 (DFE = 15) | 28.56  (DFE = 47.6) |
| Folic Acid | µg | 10.53 | 11.79 |  |  |
| Vitamin B12 | µg | 0.26 | 0.26 | 0.1 | 0.5 |
| Biotin | µg | 2.63 | 3.9 | 1 | 20 |
| Pantothenic acid | mg | 0.79 | 0.6 | 0.4 | 2 |
| Choline | mg | **15.79! (-37%)** | 31.29 | 25 | 50 |
| Calcium * | mg | 98.68 | 87.86 | 50 | 250 |
| Chromium | µg | 5.26 | 3 | / | 10 |
| Copper | mg | 0.07 | 0.09 | 0.06 | 0.12 |
| Iodine | µg | **9.21! (-39%)** | 21 | 15 | 35 |
| Iron | mg | 1.32 | 1.7 | 0.3 | 2.5 |
| Magnesium | mg | 10.53 | 12.5 | 5 | 15 |
| Manganese | mg | 0.08 | 0.006 | 0.001 | 0.1 |
| Molybdenum | µg | 5.26 | 2.6 | / | 14 |
| Phosphorus | mg | 65.79 | 64.4 | 25 | 100 |
| Selenium | µg | **2.63! (-12%)** | 3.8 | 3 | 8.6 |
| Zinc | mg | 1.05 | 1.2 | 0.5 | 2.4 |
| Potassium | mg | 98.68 | 108.29 | 80 | 160 |
| Sodium | mg | 51.32 | 41 | 25 | 60 |
| Chloride | mg | 75 | 76.14 | 60 | 160 |
| Inositol | mg | ND | 21 | 4 | 40 |
| Myo-inositol | mg | 10.53 | ND |  |  |
| Lutein | mg | ND | 0 |  |  |
| Fluoride | mg | 0.03 | 0 | / | 0.2 |
| Salt | g | 0.13 | 0.1 |  |  |

^ FOS and GOS may be added to infant formula, in which case their content shall not exceed 0.8 g/100 mL with a proportion of 90% oligogalactosyl-lactose and 10% high-molecular-weight oligofructosyl-saccharose (Commission Delegated Regulation (EU) 2016/127 of 25 September 2015). * The calcium: phosphorus molar ratio shall not be less than 1 nor greater than 2. # If added, the total concentration of nucleotides shall not exceed 5 mg/100 kcal. $ Dietary folate equivalent: 1 μg DFE = 1 μg food folate = 0.6 μg folic acid from food for special medical purposes. **Explanation of infant protein substitutes:** Infant protein substitute 2.1: MamoXi Zero Vil Infant Mix; Infant protein substitute 2.2: Nutricia MSUD Anamix Infant.

**Supplementary Table 4a.** Food components per 100 mL of infant protein substitute for GA1. If nutritional data were not present on the nutritional label, the term “Not Declared” (ND) was used.

| **Content per 100 mL** | **Unit** | **Infant Protein Substitute 3.1** | **Infant Protein Substitute 3.2** |
| --- | --- | --- | --- |
| Energy | Kj | 319 | 293 |
| Energy | Kcal | 76 | 70 |
| Total fats | g | 4 | 3.5 |
| Saturated fatty acids | g | 0.8 | 1.2 |
| Monounsaturated fatty acids | g | 1.6 | 1.7 |
| Polyunsaturated fatty acids | g | 1.7 | 0.6 |
| Caproic acid | mg | 0 | 3.98 |
| Caprylic acid | mg | 0 | 59.8 |
| Capric acid | mg | 0 | 48.3 |
| Lauric acid | mg | 0 | 369 |
| Myristic acid | mg | 10 | 160 |
| Palmitic acid | mg | 590 | 241 |
| Palmitoleic acid | mg | 10 | 5.11 |
| Stearic acid | mg | 110 | 158 |
| Oleic acid | mg | 1560 | 1604 |
| Linoleic acid (LA) | g | 1.41 | 481 |
| Alpha-linolenic acid (ALA) | g | 0.30 | 47.6 |
| Docosahexaenoic acid (DHA) | g (mg) | 0.02 (17) | 0.02 (17.8) |
| Arachidonic acid (ARA) | g (mg) | 0 | 0.02 (17.8) |
| Eicosapentaenoic acid (EPA) | mg | 3 | 0.05 |
| Arachidic acid | g | 0.07 | ND |
| Behenic acid | g | 0.02 | ND |
| Cholesterol | g | ND | 0 |
| Carbohydrates | g | 8 | 7.5 |
| Soluble carbohydrates | g | 4 | 1.1 |
| Starch | g | 1.2 | 0 |
| Polyols | g | ND | ND |
| Fructose | g | ND | 0 |
| Maltodextrins | g | 3 | ND |
| Lactose | g | 3.5 | 0.25 |
| D-mannose | g | ND | ND |
| Dietary fibre | g | 0 | 0.56 |
| 2’-Fucosyllactose | g | ND | 0 |
| Lacto-N-tetraose | g | ND | 0 |
| GOS | g | ND | 0.48 |
| FOS | g | ND | 0.08 |
| Protein equivalents | g | 1.7 | 2 |
| L-Alanine | g | 0.08 | 0.1 |
| L-Arginine | g | 0.16 | 0.18 |
| L-Aspartic acid | g | 0.23 | 0.15 |
| L-Cystine | g | 0.05 | 0.07 |
| L-Glutamine | g | 0.08 | 0.02 |
| Glycine | g | 0.13 | 0.16 |
| L-Histidine | g | 0.06 | 0.1 |
| L-Isoleucine | g | 0.12 | 0.16 |
| L-Leucine | g | 0.2 | 0.27 |
| L-Lysine | g | 0 | 0 |
| L-Methionine | g | 0.03 | 0.04 |
| L-Phenylalanine | g | 0.08 | 0.12 |
| L-Proline | g | 0.17 | 0.19 |
| L-Serine | g | 0.11 | 0.12 |
| Taurine | mg | 6 | 4.5 |
| L-Threonine | g | 0.09 | 0.13 |
| L-Tryptophan | g | 0.01 | 0.01 |
| L-Tyrosine | g | 0.18 | 0.12 |
| L-Valine | g | 0.12 | 0.17 |
| L-Carnitine | mg | 1.6 | 1.52 |
| L-Glutamic acid | g | 0.14 | 0.2 |
| Nucleotides | mg | ND | 0 |
| Vitamin A | µg RE | 86 | 61.2 |
| Vitamin D3 | µg | 1 | 1.68 |
| Vitamin E | mg α-TE | 0.7 | 1.38 |
| Vitamin C | mg | 9 | 7.34 |
| Vitamin K1 | µg | 3.6 | 5.59 |
| Vitamin K2 (MK-7) | µg | ND | ND |
| Vitamin B1, Thiamin | mg | 0.07 | 0.08 |
| Vitamin B2, Riboflavin | mg | 0.08 | 0.08 |
| Vitamin B3, Niacin | mg | 0.9 | 0.35 |
| Vitamin B6 | mg | 0.08 | 0.08 |
| Total folate | µg | ND | 13.8 |
| Folic Acid | µg | 8 | 8.25 |
| Vitamin B12 | µg | 0.2 | 0.18 |
| Biotin | µg | 2 | 2.7 |
| Pantothenic acid | mg | 0.6 | 0.42 |
| Choline | mg | 12 | 21.9 |
| Calcium | mg | 75 | 61.5 |
| Chromium | µg | 4 | 2.1 |
| Copper | mg | 0.05 | 0.06 |
| Iodine | µg | 7 | 14.7 |
| Iron | mg | 1 | 1.19 |
| Magnesium | mg | 8 | 8.75 |
| Manganese | mg | 0.06 | 0.004 |
| Molybdenum | µg | 4 | 1.82 |
| Phosphorus | mg | 50 | 45 |
| Selenium | µg | 2 | 2.66 |
| Zinc | mg | 0.8 | 0.84 |
| Potassium | mg | 75 | 75.8 |
| Sodium | mg | 39 | 28.7 |
| Chloride | mg | 57 | 53.3 |
| Inositol | mg | ND | 14.7 |
| Myo-inositol | mg | 8 | ND |
| Lutein | mg | ND | ND |
| Fluoride | mg | 0.02 | 0 |
| Salt | g | 0.1 | 0.07 |

**Explanation of infant protein substitutes:** Infant protein substitute 3.1: MamoXi Zero LYS Infant Mix^LCP^; Infant protein substitute 3.2: Nutricia GA1 Anamix Infant.

**Supplementary Table 4b.** Food components per 100 kcal of infant protein substitute compared to the Commission Delegated Regulation (EU) 2016/127 of 25 September 2015 and to the Commission Delegated Regulation (EU) 2016/128 of 25 September 2015 for vitamins and minerals. If nutritional data were not present on the nutritional label, the term “Not Declared” (ND) was used. A “!” is used when the value does not comply with the Regulation.

| **Content per 100 mL** | **Unit** | **Infant Protein Substitute 3.1** | **Infant Protein Substitute 3.2** | **MIN**  **EU 2016/127** | **MAX**  **EU 2016/127** |
| --- | --- | --- | --- | --- | --- |
|  |  | From birth | From birth |  |  |
| Energy | Kj | **319! (+9%)** | 293 | 250 | 293 |
| Energy | Kcal | **76! (+9%)** | 70 | 60 | 70 |
| **Content per 100 kcal** | **unit** | **Infant protein substitute 3.1** | **Infant protein substitute 3.2** | **MIN**  **EU 2016/127** | **MAX**  **EU 2016/127** |
| Total fats | g | 5.26 | 5 | 4.4 | 6.0 |
| Saturated fatty acids | g | 1.05 | 1.71 |  |  |
| Monounsaturated fatty acids | g | 2.11 | 2.43 |  |  |
| Polyunsaturated fatty acids | g | 2.24 | 0.86 |  |  |
| Caproic acid | mg | 0 | 5.7 |  |  |
| Caprylic acid | mg | 0 | 85.5 |  |  |
| Capric acid | mg | 0 | 69.1 |  |  |
| Lauric acid | mg | 0 | 527 |  |  |
| Myristic acid | mg | 10 | 228 |  |  |
| Palmitic acid | mg | 780 | 344 |  |  |
| Palmitoleic acid | mg | 10 | 3.7 |  |  |
| Stearic acid | mg | 140 | 226 |  |  |
| Oleic acid | mg | 2050 | 2295 |  |  |
| Linoleic acid | g | **1.86! (+55%)** | 0.69 | 0.5 | 1.2 |
| Alpha-linolenic acid | g | **0.39! (+290%)** | 0.07 | 0.05 | 0.1 |
| Docosahexaenoic acid (DHA) | g (mg) | 0.02 (22.37) | 0.03 (25.5) | 0.02 (20) | 0.05 (50) |
| Arachidonic acid (ARA) | g (mg) | 0 | 0.03 (25.5) |  |  |
| Eicosapentaenoic acid (EPA) | mg | 4 | 0.07 |  |  |
| Arachidic acid | g | 0.09 | ND |  |  |
| Behenic acid | g | 0.03 | ND |  |  |
| Cholesterol | g | ND | 0 |  |  |
| **Content per 100 kcal** | **Unit** | **Infant protein substitute 3.1** | **Infant protein substitute 3.2** | **MIN**  **EU 2016/127** | **MAX**  **EU 2016/127** |
| Carbohydrates | g | 10.53 | 10.8 | 9 | 14 |
| Soluble carbohydrates | g | 5.26 | 1.57 |  |  |
| Starch | g | 1.58 | 0 | / | 2 |
| Polyols | g | ND | ND |  |  |
| Fructose | g | ND | 0 |  |  |
| Maltodextrins | g | 3.95 | ND |  |  |
| Lactose | g | 4.61 | **0.36! (-92%)** | 4.5 | / |
| D-mannose | g | ND | ND |  |  |
| Dietary fibre | g | 0 | 0.8 |  |  |
| 2’-Fucosyllactose | g | ND | 0 |  |  |
| Lacto-N-tetraose | g | ND | 0 |  |  |
| GOS | g | ND | 0.69 |  | ^ |
| FOS | g | ND | 0.11 |  | ^ |
| **Content per 100 mL** | **Unit** | **Infant protein substitute 3.1** | **Infant protein substitute 3.2** |  |  |
| GOS | g | ND | 0.48 |  | ^ |
| FOS | g | ND | 0.08 |  | ^ |
| **Content per 100 kcal** | **Unit** | **Infant protein substitute 3.1** | **Infant protein substitute 3.2** | **MIN**  **EU 2016/127** | **MAX**  **EU 2016/127** |
| Protein equivalents | g | 2.24 | 2.86 |  |  |
| **Content per 100 kcal** | **Unit** | **Infant protein substitute 3.1** | **Infant protein substitute 3.2** | **MIN**  **EU 2016/127** | **MAX**  **EU 2016/127** |
| L-Alanine | g | 0.11 | 0.14 |  |  |
| L-Arginine | g | 0.21 | 0.26 |  |  |
| L-Aspartic acid | g | 0.3 | 0.21 |  |  |
| L-Cystine | g | 0.07 | 0.1 |  |  |
| L-Glutamine | g | 0.11 | 0.03 |  |  |
| Glycine | g | 0.17 | 0.23 |  |  |
| L-Histidine | g | 0.08 | 0.14 |  |  |
| L-Isoleucine | g | 0.16 | 0.23 |  |  |
| L-Leucine | g | 0.26 | 0.39 |  |  |
| L-Lysine | g | 0 | 0 |  |  |
| L-Methionine | g | 0.04 | 0.06 |  |  |
| L-Phenylalanine | g | 0.11 | 0.17 |  |  |
| L-Proline | g | 0.22 | 0.27 |  |  |
| L-Serine | g | 0.14 | 0.17 |  |  |
| Taurine | mg | 7.89 | 6.43 |  | 12 |
| L-Threonine | g | 0.12 | 0.19 |  |  |
| L-Tryptophan | g | 0.01 | 0.01 |  |  |
| L-Tyrosine | g | 0.24 | 0.17 |  |  |
| L-Valine | g | 0.16 | 0.24 |  |  |
| L-Carnitine | mg | 2.11 | 2.17 | 1.2 |  |
| L-Glutamic acid | g | 0.18 | 0.285 |  |  |
| Nucleotides # | mg | ND | 0 |  | 5 |
| **Content per 100 kcal** | **Unit** | **Infant protein substitute 3.1** | **Infant protein substitute 3.2** | **MIN**  **EU 2016/127** | **MAX**  **EU 2016/127** |
| Vitamin A | µg RE | 113.16 | 87.43 | 70 | 180 |
| Vitamin D3 | µg | **1.32! (-34%)** | 2.4 | 2 | 3 |
| Vitamin E | mg α-TE | 0.92 | 1.97 | 0.6 | 5 |
| Vitamin C | mg | 11.84 | 10.49 | 4 | 30 |
| Vitamin K1 | µg | 4.74 | 7.99 | 1 | 25 |
| Vitamin K2 (MK-7) | µg | ND | ND |  |  |
| Vitamin B1, Thiamin | mg | 0.09 | 0.11 | 0.04 | 0.3 |
| Vitamin B2, Riboflavin | mg | 0.11 | 0.11 | 0.06 | 0.45 |
| Vitamin B3, Niacin | mg | 1.18 | 0.5 | 0.4 | 3 |
| Vitamin B6 | mg | 0.11 | 0.11 | 0.02 | 0.3 |
| Total folate | µg | ND | 19.71 | 9 (DFE = 15) | 28.56  (DFE = 47.6) |
| Folic Acid | µg | 10.53 | 11.79 |  |  |
| Vitamin B12 | µg | 0.26 | 0.26 | 0.1 | 0.5 |
| Biotin | µg | 2.63 | 3.86 | 1 | 20 |
| Pantothenic acid | mg | 0.79 | 0.6 | 0.4 | 2 |
| Choline | mg | **15.79! (-37%)** | 31.29 | 25 | 50 |
| Calcium * | mg | 98.68 | 87.86 | 50 | 250 |
| Chromium | µg | 5.26 | 3 | / | 10 |
| Copper | mg | 0.07 | 0.09 | 0.06 | 0.12 |
| Iodine | µg | **9.21! (-39%)** | 21 | 15 | 35 |
| Iron | mg | 1.32 | 1.7 | 0.3 | 2.5 |
| Magnesium | mg | 10.53 | 12.5 | 5 | 15 |
| Manganese | mg | 0.08 | 0.006 | 0.001 | 0.1 |
| Molybdenum | µg | 5.26 | 2.6 | / | 14 |
| Phosphorus | mg | 65.79 | 64.4 | 25 | 100 |
| Selenium | µg | **2.63! (-12%)** | 3.8 | 3 | 8.6 |
| Zinc | mg | 1.05 | 1.2 | 0.5 | 2.4 |
| Potassium | mg | 98.68 | 108.29 | 80 | 160 |
| Sodium | mg | 51.32 | 41 | 25 | 60 |
| Chloride | mg | 75 | 76.14 | 60 | 160 |
| Inositol | mg | ND | 21 | 4 | 40 |
| Myo-inositol | mg | 10.53 | ND |  |  |
| Lutein | mg | ND | ND |  |  |
| Fluoride | mg | 0.03 | 0 | / | 0.2 |
| Salt | g | 0.13 | 0.1 |  |  |

^ FOS and GOS may be added to infant formula, in which case their content shall not exceed 0.8 g/100 mL with a proportion of 90% oligogalactosyl-lactose and 10% high-molecular-weight oligofructosyl-saccharose (Commission Delegated Regulation (EU) 2016/127 of 25 September 2015). * The calcium: phosphorus molar ratio shall not be less than 1 nor greater than 2. # If added, the total concentration of nucleotides shall not exceed 5 mg/100 kcal. $ Dietary folate equivalent: 1 μg DFE = 1 μg food folate = 0.6 μg folic acid from food for special medical purposes. **Explanation of infant protein substitutes:** Infant protein substitute 3.1: MamoXi Zero LYS Infant Mix; Infant protein substitute 3.2: Nutricia GA1 Anamix Infant.

**Supplementary Table 5a.** Food components per 100 mL of infant protein substitute for Classical HCU. If nutritional data were not present on the nutritional label, the term “Not Declared” (ND) was used.

| **Content per 100 mL** | **Unit** | **Infant Protein Substitute 4.1** | **Infant Protein Substitute 4.2** |
| --- | --- | --- | --- |
| Energy | Kj | 320 | 293 |
| Energy | kcal | 76 | 70 |
| Total fats | g | 4 | 3.5 |
| Saturated fatty acids | g | 0.8 | 1.2 |
| Monounsaturated fatty acids | g | 1.6 | 1.7 |
| Polyunsaturated fatty acids | g | 1.7 | 0.6 |
| Caproic acid | mg | 0 | 3.98 |
| Caprylic acid | mg | 0 | 59.8 |
| Capric acid | mg | 0 | 48.3 |
| Lauric acid | mg | 0 | 369 |
| Myristic acid | mg | 10 | 160 |
| Palmitic acid | mg | 590 | 241 |
| Palmitoleic acid | mg | 10 | 2.59 |
| Stearic acid | mg | 110 | 158 |
| Oleic acid | mg | 1560 | 1604 |
| Linoleic acid (LA) | g | 1.41 | 481 |
| Alpha-linolenic acid (ALA) | g | 0.3 | 47.6 |
| Docosahexaenoic acid (DHA) | g (mg) | 0.02 (17) | 0.02 (17.8) |
| Arachidonic acid (ARA) | g (mg) | 0 | 0.02 (17.8) |
| Eicosapentaenoic acid (EPA) | mg | 3 | 0.05 |
| Arachidic acid | g | 0.07 | ND |
| Behenic acid | g | 0.02 | ND |
| Cholesterol | g | ND | 0 |
| Carbohydrates | g | 8 | 7.5 |
| Soluble carbohydrates | g | 4 | 1.1 |
| Starch | g | 1.2 | 0 |
| Polyols | g | ND | ND |
| Fructose | g | ND | 0 |
| Maltodextrins | g | 3 | ND |
| Lactose | g | 3.5 | 0.25 |
| D-mannose | g | ND | ND |
| Dietary fibre | g | 0 | 0.56 |
| 2’-Fucosyllactose | g | ND | 0 |
| Lacto-N-tetraose | g | ND | 0 |
| GOS | g | ND | 0.48 |
| FOS | g | ND | 0.08 |
| Protein equivalents | g | 1.7 | 2 |
| L-Alanine | g | 0.08 | 0.1 |
| L-Arginine | g | 0.12 | 0.16 |
| L-Aspartic acid | g | 0.20 | 0.14 |
| L-Cystine | g | 0.07 | 0.06 |
| L-Glutamine | g | ND | 0.02 |
| Glycine | g | 0.06 | 0.15 |
| L-Histidine | g | 0.09 | 0.09 |
| L-Isoleucine | g | 0.12 | 0.15 |
| L-Leucine | g | 0.2 | 0.25 |
| L-Lysine | g | 0.14 | 0.17 |
| L-Methionine | g | 0 | 0 |
| L-Phenylalanine | g | 0.09 | 0.11 |
| L-Proline | g | 0.17 | 0.18 |
| L-Serine | g | 0.11 | 0.11 |
| Taurine | mg | 6 | 4.5 |
| L-Threonine | g | 0.09 | 0.12 |
| L-Tryptophan | g | 0.05 | 0.05 |
| L-Tyrosine | g | 0.10 | 0.11 |
| L-Valine | g | 0.12 | 0.16 |
| L-Carnitine | mg | 1.6 | 1.52 |
| L-Glutamic acid | g | 0.27 | 0.18 |
| Nucleotides | mg | ND | 0 |
| Vitamin A | µg RE | 86 | 61.2 |
| Vitamin D3 | µg | 1 | 1.68 |
| Vitamin E | mg α-TE | 0.7 | 1.38 |
| Vitamin C | mg | 9 | 7.34 |
| Vitamin K1 | µg | 3.6 | 5.59 |
| Vitamin K2 (MK-7) | µg | ND | ND |
| Vitamin B1, Thiamin | mg | 0.07 | 0.08 |
| Vitamin B2, Riboflavin | mg | 0.08 | 0.08 |
| Vitamin B3, Niacin | mg | 0.9 | 0.35 |
| Vitamin B6 | mg | 0.08 | 0.08 |
| Total folate | µg | ND | 13.8 |
| Folic Acid | µg | 8 | 8.25 |
| Vitamin B12 | µg | 0.2 | 0.18 |
| Biotin | µg | 2 | 2.7 |
| Pantothenic acid | mg | 0.6 | 0.42 |
| Choline | mg | 12 | 21.9 |
| Calcium | mg | 75 | 61.5 |
| Chromium | µg | 4 | 2.1 |
| Copper | mg | 0.05 | 0.06 |
| Iodine | µg | 7 | 14.7 |
| Iron | mg | 1 | 1.19 |
| Magnesium | mg | 8 | 8.75 |
| Manganese | mg | 0.06 | 0.004 |
| Molybdenum | µg | 4 | 1.82 |
| Phosphorus | mg | 50 | 45 |
| Selenium | µg | 2 | 2.66 |
| Zinc | mg | 0.8 | 0.84 |
| Potassium | mg | 75 | 75.8 |
| Sodium | mg | 39 | 28.7 |
| Chloride | mg | 57 | 53.3 |
| Inositol | mg | ND | 14.7 |
| Myo-inositol | mg | 8 | ND |
| Lutein | mg | ND | ND |
| Fluoride | mg | 0.02 | 0 |
| Salt | g | 0.1 | 0.07 |

**Explanation of infant protein substitutes:** Infant protein substitute 4.1: MamoXi Zero Met Infant Mix^LCP^; Infant protein substitute 4.2: Nutricia Anamix Infant HCU.

**Supplementary Table 5b.** Food components per 100 kcal of infant protein substitute for HCU compared to the Commission Delegated Regulation (EU) 2016/127 of 25 September 2015 and to the Commission Delegated Regulation (EU) 2016/128 of 25 September 2015 for vitamins and minerals. If nutritional data were not present on the nutritional label, the term “Not Declared” (ND) was used. A “!” is used when the value does not comply with the Regulation.

| **Content per 100 mL** | **Unit** | **Infant Protein Substitute 4.1** | **Infant Protein Substitute 4.2** | **MIN**  **EU 2016/127** | **MAX**  **EU 2016/127** |
| --- | --- | --- | --- | --- | --- |
|  |  | From birth | From birth |  |  |
| Energy | Kj | **320! (+9%)** | 293 | 250 | 293 |
| Energy | Kcal | **76! (+9%)** | 70 | 60 | 70 |
| **Content per 100 kcal** | **unit** | **Infant protein substitute 4.1** | **Infant protein substitute 4.2** | **MIN**  **EU 2016/127** | **MAX**  **EU 2016/127** |
| Total fats | g | 5.26 | 5 | 4.4 | 6.0 |
| Saturated fatty acids | g | 1.05 | 1.71 |  |  |
| Monounsaturated fatty acids | g | 2.11 | 2.43 |  |  |
| Polyunsaturated fatty acids | g | 2.24 | 0.86 |  |  |
| Caproic acid | mg | 0 | 5.7 |  |  |
| Caprylic acid | mg | 0 | 85.5 |  |  |
| Capric acid | mg | 0 | 69.1 |  |  |
| Lauric acid | mg | 0 | 527 |  |  |
| Myristic acid | mg | 10 | 228 |  |  |
| Palmitic acid | mg | 780 | 344 |  |  |
| Palmitoleic acid | mg | 10 | 3.7 |  |  |
| Stearic acid | mg | 140 | 226 |  |  |
| Oleic acid | mg | 2050 | 2295 |  |  |
| Linoleic acid | g | **1.86! (+55%)** | 0.69 | 0.5 | 1.2 |
| Alpha-linolenic acid | g | **0.39! (+290%)** | 0.07 | 0.05 | 0.1 |
| Docosahexaenoic acid (DHA) | g (mg) | 0.02 (22.37) | 0.03 (25.5) | 0.02 (20) | 0.05 (50) |
| Arachidonic acid (ARA) | g (mg) | 0 | 0.03 (25.5) |  |  |
| Eicosapentaenoic acid (EPA) | mg | 4 | 0.07 |  |  |
| Arachidic acid | g | 0.09 | ND |  |  |
| Behenic acid | g | 0.03 | ND |  |  |
| Cholesterol | g | ND | 0 |  |  |
| **Content per 100 kcal** | **Unit** | **Infant protein substitute 4.1** | **Infant protein substitute 4.2** | **MIN**  **EU 2016/127** | **MAX**  **EU 2016/127** |
| Carbohydrates | g | 10.53 | 10.8 | 9 | 14 |
| Soluble carbohydrates | g | 5.26 | 1.57 |  |  |
| Starch | g | 1.58 | 0 | / | 2 |
| Polyols | g | ND | ND |  |  |
| Fructose | g | ND | 0 |  |  |
| Maltodextrins | g | 3.95 | ND |  |  |
| Lactose | g | 4.61 | **0.36! (-92%)** | 4.5 | / |
| D-mannose | g | ND | ND |  |  |
| Dietary fibre | g | 0 | 0.8 |  |  |
| 2’-Fucosyllactose | g | ND | 0 |  |  |
| Lacto-N-tetraose | g | ND | 0 |  |  |
| GOS | g | ND | 0.69 |  | ^ |
| FOS | g | ND | 0.11 |  | ^ |
| **Content per 100 mL** | **Unit** | **Infant protein substitute 4.1** | **Infant protein substitute 4.2** |  |  |
| GOS | g | ND | 0.48 |  | ^ |
| FOS | g | ND | 0.08 |  | ^ |
| **Content per 100 kcal** | **Unit** | **Infant protein substitute 4.1** | **Infant protein substitute 4.2** | **MIN**  **EU 2016/127** | **MAX**  **EU 2016/127** |
| Protein equivalents | g | 2.24 | 2.86 |  |  |
| **Content per 100 kcal** | **Unit** | **Infant protein substitute 4.1** | **Infant protein substitute 4.2** | **MIN**  **EU 2016/127** | **MAX**  **EU 2016/127** |
| L-Alanine | g | 0.11 | 0.14 |  |  |
| L-Arginine | g | 0.16 | 0.23 |  |  |
| L-Aspartic acid | g | 0.26 | 0.2 |  |  |
| L-Cystine | g | 0.09 | 0.09 |  |  |
| L-Glutamine | g | ND | 0.03 |  |  |
| Glycine | g | 0.08 | 0.21 |  |  |
| L-Histidine | g | 0.12 | 0.13 |  |  |
| L-Isoleucine | g | 0.16 | 0.21 |  |  |
| L-Leucine | g | 0.26 | 0.36 |  |  |
| L-Lysine | g | 0.18 | 0.24 |  |  |
| L-Methionine | g | 0 | 0 |  |  |
| L-Phenylalanine | g | 0.12 | 0.16 |  |  |
| L-Proline | g | 0.22 | 0.26 |  |  |
| L-Serine | g | 0.14 | 0.16 |  |  |
| Taurine | mg | 7.89 | 6.43 |  | 12 |
| L-Threonine | g | 0.12 | 0.17 |  |  |
| L-Tryptophan | g | 0.07 | 0.07 |  |  |
| L-Tyrosine | g | 0.13 | 0.16 |  |  |
| L-Valine | g | 0.16 | 0.23 |  |  |
| L-Carnitine | mg | 2.11 | 2.17 | 1.2 |  |
| L-Glutamic acid | g | 0.36 | 0.26 |  |  |
| Nucleotides # | mg | ND | 0 |  | 5 |
| **Content per 100 kcal** | **Unit** | **Infant protein substitute 4.1** | **Infant protein substitute 4.2** | **MIN**  **EU 2016/127** | **MAX**  **EU 2016/127** |
| Vitamin A | µg RE | 113.16 | 87.43 | 70 | 180 |
| Vitamin D3 | µg | **1.32! (-34%)** | 2.4 | 2 | 3 |
| Vitamin E | mg α-TE | 0.92 | 1.97 | 0.6 | 5 |
| Vitamin C | mg | 11.84 | 10.49 | 4 | 30 |
| Vitamin K1 | µg | 4.74 | 7.99 | 1 | 25 |
| Vitamin K2 (MK-7) | µg | ND | ND |  |  |
| Vitamin B1, Thiamin | mg | 0.09 | 0.11 | 0.04 | 0.3 |
| Vitamin B2, Riboflavin | mg | 0.11 | 0.11 | 0.06 | 0.45 |
| Vitamin B3, Niacin | mg | 1.18 | 0.5 | 0.4 | 3 |
| Vitamin B6 | mg | 0.11 | 0.11 | 0.02 | 0.3 |
| Total folate $ | µg | ND | 19.71 | 9 (DFE = 15) | 28.56  (DFE = 47.6) |
| Folic Acid | µg | 10.53 | 11.79 |  |  |
| Vitamin B12 | µg | 0.26 | 0.26 | 0.1 | 0.5 |
| Biotin | µg | 2.63 | 3.86 | 1 | 20 |
| Pantothenic acid | mg | 0.79 | 0.6 | 0.4 | 2 |
| Choline | mg | **15.79! (-37%)** | 31.29 | 25 | 50 |
| Calcium * | mg | 98.68 | 87.86 | 50 | 250 |
| Chromium | µg | 5.26 | 3 | / | 10 |
| Copper | mg | 0.07 | 0.09 | 0.06 | 0.12 |
| Iodine | µg | **9.21! (-39%)** | 21 | 15 | 35 |
| Iron | mg | 1.32 | 1.7 | 0.3 | 2.5 |
| Magnesium | mg | 10.53 | 12.5 | 5 | 15 |
| Manganese | mg | 0.08 | 0.006 | 0.001 | 0.1 |
| Molybdenum | µg | 5.26 | 2.6 | / | 14 |
| Phosphorus | mg | 65.79 | 64.29 | 25 | 100 |
| Selenium | µg | **2.63! (-12%)** | 3.8 | 3 | 8.6 |
| Zinc | mg | 1.05 | 1.2 | 0.5 | 2.4 |
| Potassium | mg | 98.68 | 108.29 | 80 | 160 |
| Sodium | mg | 51.32 | 41 | 25 | 60 |
| Chloride | mg | 75 | 76.14 | 60 | 160 |
| Inositol | mg | ND | 21 | 4 | 40 |
| Myo-inositol | mg | 10.53 | ND |  |  |
| Lutein | mg | ND | ND |  |  |
| Fluoride | mg | 0.03 | 0 | / | 0.2 |
| Salt | g | 0.13 | 0.1 |  |  |

^ FOS and GOS may be added to infant formula, in which case their content shall not exceed 0.8 g/100 mL with a proportion of 90% oligogalactosyl-lactose and 10% high-molecular-weight oligofructosyl-saccharose (Commission Delegated Regulation (EU) 2016/127 of 25 September 2015). * The calcium: phosphorus molar ratio shall not be less than 1 nor greater than 2. # If added, the total concentration of nucleotides shall not exceed 5 mg/100 kcal. $ Dietary folate equivalent: 1 μg DFE = 1 μg food folate = 0.6 μg folic acid from food for special medical purposes. **Explanation of infant protein substitutes:** Infant protein substitute 4.1: MamoXi Zero Met Infant Mix^LCP^; Infant protein substitute 4.2: Nutricia Anamix Infant HCU.

**Supplementary Table 6a.** Food components per 100 mL of infant protein substitute for classical organic acidurias (Infant Protein Substitute 5.1 for IVA; Infant Protein Substitutes 5.2 and 5.3 for PA and MMA). If nutritional data were not present on the nutritional label, the term “Not Declared” (ND) was used.

| **Content per 100 mL** | **Unit** | **Infant Protein Substitute 5.1** | **Infant Protein Substitute 5.2** | **Infant Protein Substitute 5.3** |
| --- | --- | --- | --- | --- |
| Energy | Kj | 293 | 319 | 293 |
| Energy | Kcal | 70 | 76 | 70 |
| Total fats | g | 3.5 | 4 | 3.5 |
| Saturated fatty acids | g | 1.2 | 0.8 | 1.2 |
| Monounsaturated fatty acids | g | 1.7 | 1.6 | 1.7 |
| Polyunsaturated fatty acids | g | 0.6 | 1.7 | 0.6 |
| Caproic acid | mg | ND | 0 | ND |
| Caprylic acid | mg | 3.98 | 0 | 3.98 |
| Capric acid | mg | 59.8 | 0 | 59.8 |
| Lauric acid | mg | 48.3 | 0 | 48.3 |
| Myristic acid | mg | 160 | 0.01 | 160 |
| Palmitic acid | mg | 241 | 0.59 | 241 |
| Palmitoleic acid | mg | 2.59 | 0.01 | 2.59 |
| Stearic acid | mg | 158 | 0.11 | 158 |
| Oleic acid | mg | 1604 | 1.56 | 1604 |
| Linoleic acid (LA) | g | 481 | 1.41 | 481 |
| Alpha-linolenic acid (ALA) | g | 47.6 | 0.30 | 47.6 |
| Docosahexaenoic acid (DHA) | g (mg) | 0.02 (17.8) | 0.02 (17) | 0.02 (17.8) |
| Arachidonic acid (ARA) | g (mg) | 0.02 (17.8) | 0 | 0.02 (17.8) |
| Eicosapentaenoic acid (EPA) | mg | 0.05 | 3 | 0.05 |
| Arachidic acid | g | ND | 0.07 | ND |
| Behenic acid | g | ND | 0.02 | ND |
| Cholesterol | g | 0 | ND | 0 |
| Carbohydrates | g | 7.5 | 8 | 7.5 |
| Soluble carbohydrates | g | 1.1 | 3.5 | 1.1 |
| Starch | g | 0 | 1.2 | 0 |
| Polyols | g | ND | ND | ND |
| Fructose | g | 0 | ND | 0 |
| Maltodextrins | g | ND | 3 | ND |
| Lactose | g | 0.25 | 3.5 | 0.25 |
| D-mannose | g | ND | ND | ND |
| Dietary fibre | g | 0.56 | 0 | 0.56 |
| 2’-Fucosyllactose | g | 0 | ND | 0 |
| Lacto-N-tetraose | g | 0 | ND | 0 |
| GOS | g | 0.48 | ND | 0.48 |
| FOS | g | 0.08 | ND | 0.08 |
| Protein equivalents | g | 2 | 1.7 | 2 |
| L-Alanine | g | 0.11 | 0.12 | 0.24 |
| L-Arginine | g | 0.18 | 0.14 | 0.21 |
| L-Aspartic acid | g | 0.15 | 0.21 | 0.19 |
| L-Cystine | g | 0.07 | 0.07 | 0.08 |
| L-Glutamine | g | 0.02 | ND | 0.02 |
| Glycine | g | 0.33 | 0.12 | 0.1 |
| L-Histidine | g | 0.11 | 0.09 | 0.14 |
| L-Isoleucine | g | 0.06 | 0 | <0.007 |
| L-Leucine | g | 0 | 0.2 | 0.32 |
| L-Lysine | g | 0.16 | 0.18 | 0.21 |
| L-Methionine | g | 0.05 | 0 | 0 |
| L-Phenylalanine | g | 0.12 | 0.11 | 0.14 |
| L-Proline | g | 0.33 | 0.17 | 0.1 |
| L-Serine | g | 0.1 | 0.11 | 0.13 |
| Taurine | mg | 4.5 | 6 | 4.5 |
| L-Threonine | g | 0.11 | 0 | 0 |
| L-Tryptophan | g | 0.04 | 0.05 | 0.06 |
| L-Tyrosine | g | 0.12 | 0.12 | 0.14 |
| L-Valine | g | 0.07 | 0 | 0 |
| L-Carnitine | mg | 1.52 | 1.6 | 1.52 |
| L-Glutamic acid | g | 0.203 | 0.32 | 0.24 |
| Nucleotides | mg | 0 | ND | 0 |
| Vitamin A | µg RE | 61.2 | 86 | 61.2 |
| Vitamin D3 | µg | 1.68 | 1 | 1.68 |
| Vitamin E | mg α-TE | 1.38 | 0.7 | 1.38 |
| Vitamin C | mg | 7.34 | 9 | 7.34 |
| Vitamin K1 | µg | 5.59 | 3.6 | 5.59 |
| Vitamin K2 (MK-7) | µg | ND | ND | ND |
| Vitamin B1, Thiamin | mg | 0.08 | 0.07 | 0.08 |
| Vitamin B2, Riboflavin | mg | 0.08 | 0.08 | 0.08 |
| Vitamin B3, Niacin | mg | 0.35 | 0.9 | 0.35 |
| Vitamin B6 | mg | 0.08 | 0.08 | 0.08 |
| Total folate | µg | 13.8 | ND | 13.8 |
| Folic Acid | µg | 8.25 | 8 | 8.25 |
| Vitamin B12 | µg | 0.18 | 0.2 | 0.18 |
| Biotin | µg | 2.7 | 2 | 2.7 |
| Pantothenic acid | mg | 0.42 | 0.6 | 0.42 |
| Choline | mg | 21.9 | 12 | 21.9 |
| Calcium | mg | 61.5 | 75 | 61.5 |
| Chromium | µg | 2.1 | 4 | 2.1 |
| Copper | mg | 0.06 | 0.05 | 0.06 |
| Iodine | µg | 14.7 | 7 | 14.7 |
| Iron | mg | 1.19 | 1 | 1.19 |
| Magnesium | mg | 8.75 | 8 | 8.75 |
| Manganese | mg | 0.004 | 0.06 | 0.004 |
| Molybdenum | µg | 1.82 | 4 | 1.82 |
| Phosphorus | mg | 45 | 50 | 45 |
| Selenium | µg | 2.66 | 2 | 2.66 |
| Zinc | mg | 0.84 | 0.8 | 0.84 |
| Potassium | mg | 75.8 | 75 | 75.8 |
| Sodium | mg | 28.7 | 39 | 28.7 |
| Chloride | mg | 53.3 | 57 | 53.3 |
| Inositol | mg | 14.7 | ND | 14.7 |
| Myo-inositol | mg | ND | 8 | ND |
| Lutein | mg | ND | ND | ND |
| Fluoride | mg | 0 | 0.02 | 0 |
| Salt | g | 0.07 | 0.1 | 0.07 |

**Explanation of infant protein substitutes:** Infant protein substitute 5.1: Nutricia IVA Anamix Infant; Infant protein substitute 5.2: MamoXi Zero TVMI Infant Mix^LCP^; Infant protein substitute 5.3 Nutricia MMA/PA Anamix Infant.

**Supplementary** **Table 6b.** Food components per 100 kcal of infant protein substitute for organic acidurias compared to the Commission Delegated Regulation (EU) 2016/127 of 25 September 2015 and to the Commission Delegated Regulation (EU) 2016/128 of 25 September 2015 for vitamins and minerals. If nutritional data were not present on the nutritional label, the term “Not Declared” (ND) was used. A “!” is used when the value does not comply with the Regulation.

| **Content per 100 mL** | **Unit** | **Infant Protein Substitute 5.1** | **Infant Protein Substitute 5.2** | **Infant Protein Substitute 5.3** | **MIN**  **EU 2016/127** | **MAX**  **EU 2016/127** |
| --- | --- | --- | --- | --- | --- | --- |
|  |  | From birth | From birth | From birth |  |  |
| Energy | Kj | 293 | **319! (+9%)** | 293 | 250 | 293 |
| Energy | Kcal | 70 | **76! (+9%)** | 70 | 60 | 70 |
| **Content per 100 kcal** | **Unit** | **Infant protein substitute 5.2** | **Infant protein substitute 5.1** | **Infant Protein Substitute 5.3** | **MIN**  **EU 2016/127** | **MAX**  **EU 2016/127** |
| Total fats | g | 5 | 5.26 | 5 | 4.4 | 6.0 |
| Saturated fatty acids | g | 1.71 | 1.05 | 1.71 |  |  |
| Monounsaturated fatty acids | g | 2.43 | 2.11 | 2.43 |  |  |
| Polyunsaturated fatty acids | g | 0.86 | 2.24 | 0.86 |  |  |
| Caproic acid | mg | 5.7 | 0 | 5.7 |  |  |
| Caprylic acid | g | 85.5 | 0 | 85.5 |  |  |
| Capric acid | g | 69.1 | 0 | 69.1 |  |  |
| Lauric acid | g | 527 | 0 | 527 |  |  |
| Myristic acid | g | 228 | 0.01 | 228 |  |  |
| Palmitic acid | g | 344 | 0.78 | 344 |  |  |
| Palmitoleic acid | g | 3.7 | 0.01 | 3.7 |  |  |
| Stearic acid | g | 226 | 00.14 | 226 |  |  |
| Oleic acid | g | 2295 | 2.05 | 2295 |  |  |
| Linoleic acid | g | 0.69 | **1.86! (+55%)** | 0.69 | 0.5 | 1.2 |
| Alpha-linolenic acid | g | 0.07 | **0.39! (+290%)** | 0.07 | 0.05 | 0.1 |
| Docosahexaenoic acid (DHA) | g (mg) | 0.03 (25.5) | 0.02 (22.37) | 0.03 (25.5) | 0.02 (20) | 0.05 (50) |
| Arachidonic acid (ARA) | g (mg) | 0.03 (25.5) | 0 | 0.03 (25.5) |  |  |
| Eicosapentaenoic acid (EPA) | mg | 0.07 | 4 | 0.07 |  |  |
| Arachidic acid | g | ND | 0.09 | ND |  |  |
| Behenic acid | g | ND | 0.03 | ND |  |  |
| Cholesterol | g | 0 | ND | 0 |  |  |
| **Content per 100 kcal** | **Unit** | **Infant protein substitute 5.2** | **Infant protein substitute 5.1** | **Infant protein substitute 5.3** | **MIN**  **EU 2016/127** | **MAX**  **EU 2016/127** |
| Carbohydrates | g | 10.71 | 10.53 | 10.71 | 9 | 14 |
| Soluble carbohydrates | g | 1.57 | 4.61 | 1.57 |  |  |
| Starch | g | 0 | 1.58 | 0 | / | 2 |
| Polyols | g | ND | ND | ND |  |  |
| Fructose | g | 0 | ND | 0 |  |  |
| Maltodextrins | g | ND | 3.95 | ND |  |  |
| Lactose | g | **0.36! (-92%)** | 4.61 | **0.36! (-92%)** | 4.5 | / |
| D-mannose | g | ND | ND | ND |  |  |
| Dietary fibre | g | 0.8 | 0 | 0.8 |  |  |
| 2’-Fucosyllactose | g | 0 | ND | 0 |  |  |
| Lacto-N-tetraose | g | 0 | ND | 0 |  |  |
| GOS | g | 0.69 | ND | 0.69 |  | ^ |
| FOS | g | 0.11 | ND | 0.11 |  | ^ |
| **Content per 100 mL** | **Unit** | **Infant protein substitute 5.2** | **Infant protein substitute 5.1** | **Infant protein substitute 5.3** |  |  |
| GOS | g | 0.48 | ND | 0.48 |  | ^ |
| FOS | g | 0.08 | ND | 0.08 |  | ^ |
| **Content per 100 kcal** | **Unit** | **Infant protein substitute 5.2** | **Infant protein substitute 5.1** | **Infant protein substitute 5.3** | **MIN**  **EU 2016/127** | **MAX**  **EU 2016/127** |
| Protein equivalents | g | 2.86 | 2.24 | 2.86 |  |  |
| **Content per 100 kcal** | **Unit** | **Infant protein substitute 5.2** | **Infant protein substitute 5.1** | **Infant protein substitute 5.3** | **MIN**  **EU 2016/127** | **MAX**  **EU 2016/127** |
| L-Alanine | g | 0.16 | 0.16 | 0.34 |  |  |
| L-Arginine | g | 0.26 | 0.18 | 0.3 |  |  |
| L-Aspartic acid | g | 0.21 | 0.28 | 0.27 |  |  |
| L-Cystine | g | 0.1 | 0.09 | 0.11 |  |  |
| L-Glutamine | g | 0.03 | ND | 0.03 |  |  |
| Glycine | g | 0.47 | 0.16 | 0.14 |  |  |
| L-Histidine | g | 0.16 | 0.12 | 0.2 |  |  |
| L-Isoleucine | g | 0.09 | 0 | 0 |  |  |
| L-Leucine | g | 0 | 0.26 | 0.46 |  |  |
| L-Lysine | g | 0.23 | 0.24 | 0.3 |  |  |
| L-Methionine | g | 0.07 | 0 | 0 |  |  |
| L-Phenylalanine | g | 0.17 | 0.14 | 0.2 |  |  |
| L-Proline | g | 0.47 | 0.22 | 0.14 |  |  |
| L-Serine | g | 0.14 | 0.14 | 0.19 |  |  |
| Taurine | mg | 6.43 | 7.89 | 6.43 |  | 12 |
| L-Threonine | g | 0.16 | 0 | 0 |  |  |
| L-Tryptophan | g | 0.06 | 0.07 | 0.09 |  |  |
| L-Tyrosine | g | 0.17 | 0.16 | 0.2 |  |  |
| L-Valine | g | 0.1 | 0 | 0 |  |  |
| L-Carnitine | mg | 2.17 | 2.11 | 2.17 | 1.2 |  |
| L-Glutamic acid | g | 0.29 | 0.42 | 0.29 |  |  |
| Nucleotides # | mg | 0 | ND | 0 |  | 5 |
| **Content per 100 kcal** | **Unit** | **Infant protein substitute 5.2** | **Infant protein substitute 5.1** | **Infant protein substitute 5.3** | **MIN**  **EU 2016/127** | **MAX**  **EU 2016/127** |
| Vitamin A | µg RE | 87.43 | 113.16 | 87.43 | 70 | 180 |
| Vitamin D3 | µg | 2.4 | **1.32! (-34%)** | 2.4 | 2 | 3 |
| Vitamin E | mg α-TE | 1.97 | 0.92 | 1.97 | 0.6 | 5 |
| Vitamin C | mg | 10.49 | 11.84 | 10.49 | 4 | 30 |
| Vitamin K1 | µg | 7.99 | 4.74 | 7.99 | 1 | 25 |
| Vitamin K2 (MK-7) | µg | ND | ND | ND |  |  |
| Vitamin B1, Thiamin | mg | 0.11 | 0.09 | 0.11 | 0.04 | 0.3 |
| Vitamin B2, Riboflavin | mg | 0.11 | 0.11 | 0.11 | 0.06 | 0.45 |
| Vitamin B3, Niacin | mg | 0.5 | 1.18 | 0.5 | 0.4 | 3 |
| Vitamin B6 | mg | 0.11 | 0.11 | 0.11 | 0.02 | 0.3 |
| Total folate $ | µg | 19.71 | ND | 19.71 | 9 (DFE = 15) | 28.56  (DFE = 47.6) |
| Folic Acid | µg | 11.79 | 10.53 | 11.79 |  |  |
| Vitamin B12 | µg | 0.26 | 0.26 | 0.26 | 0.1 | 0.5 |
| Biotin | µg | 3.86 | 2.63 | 3.86 | 1 | 20 |
| Pantothenic acid | mg | 0.6 | 0.79 | 0.6 | 0.4 | 2 |
| Choline | mg | 31.29 | **15.79! (-37%)** | 31.29 | 25 | 50 |
| Calcium * | mg | 87.86 | 98.68 | 87.86 | 50 | 250 |
| Chromium | µg | 3 | 5.26 | 3 | / | 10 |
| Copper | mg | 0.09 | 0.07 | 0.09 | 0.06 | 0.12 |
| Iodine | µg | 21 | **9.21! (-39%)** | 21 | 15 | 35 |
| Iron | mg | 1.7 | 1.32 | 1.7 | 0.3 | 2.5 |
| Magnesium | mg | 12.5 | 10.53 | 12.5 | 5 | 15 |
| Manganese | mg | 0.006 | 0.08 | 0.006 | 0.001 | 0.1 |
| Molybdenum | µg | 2.6 | 5.26 | 2.6 | / | 14 |
| Phosphorus | mg | 64.29 | 65.79 | 64.29 | 25 | 100 |
| Selenium | µg | 3.8 | **2.63! (-12%)** | 3.8 | 3 | 8.6 |
| Zinc | mg | 1.2 | 1.05 | 1.2 | 0.5 | 2.4 |
| Potassium | mg | 108.29 | 98.68 | 108.29 | 80 | 160 |
| Sodium | mg | 41 | 51.32 | 41 | 25 | 60 |
| Chloride | mg | 76.14 | 75 | 76.14 | 60 | 160 |
| Inositol | mg | 21 | ND | 21 | 4 | 40 |
| Myo-inositol | mg | ND | 10.53 | ND |  |  |
| Lutein | mg | ND | ND | ND |  |  |
| Fluoride | mg | ND | 0.03 | ND | / | 0.2 |
| Salt | g | 0.1 | 0.13 | 0.1 |  |  |

^ FOS and GOS may be added to infant formula, in which case their content shall not exceed 0.8 g/100 mL with a proportion of 90% oligogalactosyl-lactose and 10% high-molecular-weight oligofructosyl-saccharose (Commission Delegated Regulation (EU) 2016/127 of 25 September 2015). *The calcium: available phosphorus molar ratio shall not be less than 1 nor greater than 2. # If added, the total concentration of nucleotides shall not exceed 5 mg/100 kcal. $ Dietary folate equivalent: 1 μg DFE = 1 μg food folate = 0.6 μg folic acid from food for special medical purposes. **Explanation of infant protein substitutes:** Infant protein substitute 5.1: Nutricia IVA Anamix Infant; Infant protein substitute 5.2: MamoXi Zero TVMi Infant Mix^LCP^; Infant protein substitute 5.3 Nutricia MMA/PA Anamix Infant.

**Supplementary Table 7**. Food components per 100 mL and 100 kcal of infant protein substitute for UCDs. If nutritional data were not present on the nutritional label, the term “Not Declared” (ND) was used**.**

| **Content** | **Unit** | **Infant Protein Substitute 6.1** | |
| --- | --- | --- | --- |
|  |  | **100 mL** | **100 Kcal** |
| Energy | kJ | 114 | / |
|  | kcal | 27 | / |
| Total fats | g | 0 | 0 |
| Saturated fatty acids | g | 0 | 0 |
| Monounsaturated fatty acids | g | 0 | 0 |
| Polyunsaturated fatty acids | g | 0 | 0 |
| Caproic acid | g | 0 | 0 |
| Caprylic acid | g | 0 | 0 |
| Capric acid | g | 0 | 0 |
| Lauric acid | g | 0 | 0 |
| Myristic acid | g | 0 | 0 |
| Palmitic acid | g | 0 | 0 |
| Palmitoleic acid | g | 0 | 0 |
| Stearic acid | g | 0 | 0 |
| Oleic acid | g | 0 | 0 |
| Linoleic acid | g | 0 | 0 |
| Alpha-linolenic acid | g | 0 | 0 |
| Docosahexaenic acid (DHA) | g | 0 | 0 |
| Arachidonic acid (ARA) | g | 0 | 0 |
| Eicosapentaenoic acid (EPA) | g | 0 | 0 |
| Cholesterol | g | 0 | 0 |
| Carbohydrates | g | 2.0 | 7.41 |
| Soluble carbohydrates | g | 0.07 | 0.26 |
| Starch | g | 0 | 0 |
| Polyols | g | ND | ND |
| Fructose | g | 0 | 0 |
| Maltodextrins | g | ND | ND |
| Lactose | g | 0 | 0 |
| D-Mannose | g | ND | ND |
| Dietary fibre | g | 0 | 0 |
| GOS | g | 0 | 0 |
| FOS | g | 0 | 0 |
| Protein equivalents | g | 5.0 | 18.52 |
| Salt | g | 0.26 | 0.93 |
| L-Alanine | g | 0 | 0 |
| L-Arginine | g | 0 | 0 |
| L-aspartic acid | g | 0 | 0 |
| L-Cystine | g | 0.26 | 0.96 |
| L-Glutamine | g | 0 | 0 |
| Glicine | g | 0 | 0 |
| L-Histidine | g | 0.26 | 0.96 |
| L-Isoleucine | g | 0.64 | 2.37 |
| L-Leucine | g | 1.07 | 3.96 |
| L-Lysine | g | 0.76 | 2.81 |
| L-Methionine | g | 0.26 | 0.96 |
| L-Phenylalanine | g | 0.44 | 1.63 |
| L-Proline | g | 0 | 0 |
| L-Serine | g | 0 | 0 |
| Taurine | mg | 35.3 | 130.7 |
| L-Threonine | g | 0.505 | 1.87 |
| L-Tryptophan | g | 0.185 | 0.69 |
| L-Tyrosine | g | 0.545 | 2.02 |
| L-Valine | g | 0.76 | 2.81 |
| L-Carnitine | mg | 11.3 | 41.85 |
| l-Glutamic acid | g | 0 | 0 |
| Vitamin A | µg RE | 285 | 1055.56 |
| Vitamin D3 | µg | 6.38 | 23.63 |
| Vitamin E | mg α-TE | 2.40 | 8.89 |
| Vitamin C | mg | 15.0 | 55.56 |
| Vitamin K1 | µg | 7.88 | 18.75 |
| Vitamin K2 (MK-7) | µg | ND | ND |
| Vitamin B1, Thiamin | mg | 0.30 | 1.11 |
| Vitamin B2. Riboflavin | mg | 0.34 | 1.26 |
| Vitamin B3. Niacin | mg | 3.38 | 12.52 |
| Vitamin B6 | mg | 0.26 | 0.96 |
| Folic Acid | µg | 37.5 | 138.89 |
| Vitamin B12 | µg | 0.64 | 2.37 |
| Biotin | µg | 7.5 | 27.78 |
| Pantothenic Acid | mg | 2.25 | 8.33 |
| Choline | mg | 105 | 388.89 |
| Calcium | g | 0.29 | 1.07 |
| Chromium | µg | 12 | 44.44 |
| Copper | mg | 0.27 | 1.00 |
| Iodine | µg | 45.0 | 166.67 |
| Iron | mg | 5.26 | 19.48 |
| Magnesium | g | 0.036 | 0.13 |
| Manganese | mg | 0.040 | 0.15 |
| Molybdenum | µg | 15 | 55.56 |
| Phosphorus | g | 0.172 | 0.64 |
| Selenium | µg | 9.0 | 33.33 |
| Zinc | mg | 2.48 | 9.19 |
| Potassium | g | 0.338 | 1.25 |
| Sodium | g | 0.105 | 0.39 |
| Chloride | g | 0.210 | 0.78 |
| Inositol | mg | 67.5 | 250.00 |
| Myo-inositol | mg | nd | nd |
| Lutein | mg | 0 | 0.00 |
| Fluoride | mg | 0.08 | 0.30 |

**Explanation of Infant Protein Substitute 7a:** Milupa UCD-1.

**References**

1. Morrow G. Tanguay RM. Biochemical and Clinical Aspects of Hereditary Tyrosinemia Type 1. Adv Exp Med Biol. 2017;959:9–21.

2. Chinsky JM. Singh R. Ficicioglu C. van Karnebeek CDM. Grompe M. Mitchell G. et al. Diagnosis and treatment of tyrosinemia type I: a US and Canadian consensus group review and recommendations. Genet Med. 2017 Dec;19(12).

3. Castilloux J. Laberge AM. Martin SR. Lallier M. Marchand V. ‘Silent’ tyrosinemia presenting as hepatocellular carcinoma in a 10-year-old girl. J Pediatr Gastroenterol Nutr. 2007 Mar;44(3):375–7.

4. de Laet C. Dionisi-Vici C. Leonard JV. McKiernan P. Mitchell G. Monti L. et al. Recommendations for the management of tyrosinemia type 1. Orphanet J Rare Dis. 2013 Jan 11;8:8.

5. van Spronsen FJ. van Rijn M. Meyer U. Das AM. Dietary Considerations in Tyrosinemia Type I. Adv Exp Med Biol. 2017;959:197–204.

6. Burrage LC. Nagamani SCS. Campeau PM. Lee BH. Branched-chain amino acid metabolism: from rare Mendelian diseases to more common disorders. Hum Mol Genet. 2014 Sep 15;23(R1):R1-8.

7. Levin ML. Scheimann A. Lewis RA. Beaudet AL. Cerebral edema in maple syrup urine disease. J Pediatr. 1993 Jan;122(1):167–8.

8. Strauss KA. Wardley B. Robinson D. Hendrickson C. Rider NL. Puffenberger EG. et al. Classical maple syrup urine disease and brain development: principles of management and formula design. Mol Genet Metab. 2010 Apr;99(4):333–45.

9. Thompson GN. Francis DE. Halliday D. Acute illness in maple syrup urine disease: dynamics of protein metabolism and implications for management. J Pediatr. 1991 Jul;119(1 Pt 1):35–41.

10. Frazier DM. Allgeier C. Homer C. Marriage BJ. Ogata B. Rohr F. et al. Nutrition management guideline for maple syrup urine disease: an evidence- and consensus-based approach. Mol Genet Metab. 2014 Jul;112(3):210–7.

11. van Calcar S. Nutrition Management of Maple Syrup Urine Disease. In: Nutrition Management of Inherited Metabolic Diseases [Internet]. Springer International Publishing; 2022 [cited 2024 Dec 3]. p. 241–54. Available from: http://www.scopus.com/inward/record.url?scp=85159032500&partnerID=8YFLogxK

12. Blackburn PR. Gass JM. Vairo FPE. Farnham KM. Atwal HK. Macklin S. et al. Maple syrup urine disease: mechanisms and management. Appl Clin Genet. 2017;10:57–66.

13. Boy N. Mühlhausen C. Maier EM. Ballhausen D. Baumgartner MR. Beblo S. et al. Recommendations for diagnosing and managing individuals with glutaric aciduria type 1: Third revision. J Inherit Metab Dis. 2023 May;46(3):482–519.

14. Wajner M. Amaral AU. Leipnitz G. Seminotti B. Pathogenesis of brain damage in glutaric acidemia type I: Lessons from the genetic mice model. Int J Dev Neurosci. 2019 Nov;78:215–21.

15. Kölker S. Valayannopoulos V. Burlina AB. Sykut-Cegielska J. Wijburg FA. Teles EL. et al. The phenotypic spectrum of organic acidurias and urea cycle disorders. Part 2: the evolving clinical phenotype. J Inherit Metab Dis. 2015 Nov;38(6):1059–74.

16. Boy N. Mengler K. Heringer-Seifert J. Hoffmann GF. Garbade SF. Kölker S. Impact of newborn screening and quality of therapy on the neurological outcome in glutaric aciduria type 1: a meta-analysis. Genet Med. 2021 Jan;23(1):13–21.

17. Strauss KA. Brumbaugh J. Duffy A. Wardley B. Robinson D. Hendrickson C. et al. Safety. efficacy and physiological actions of a lysine-free. arginine-rich formula to treat glutaryl-CoA dehydrogenase deficiency: focus on cerebral amino acid influx. Mol Genet Metab. 2011;104(1–2):93–106.

18. BIMDG :: British Inherited Metabolic Disease Group [Internet]. [cited 2024 Dec 3]. Available from: https://www.bimdg.org.uk/site/guidelines-enbs.asp?t=2

19. Vitoria-Miñana I. Couce ML. González-Lamuño D. García-Peris M. Correcher-Medina P. Breastfeeding and Inborn Errors of Amino Acid and Protein Metabolism: A Spreadsheet to Calculate Optimal Intake of Human Milk and Disease-Specific Formulas. Nutrients. 2023 Aug 13;15(16):3566.

20. Kumar T. Sharma GS. Singh LR. Homocystinuria: Therapeutic approach. Clin Chim Acta. 2016 Jul 1;458:55–62.

21. Morris AAM. Kožich V. Santra S. Andria G. Ben-Omran TIM. Chakrapani AB. et al. Guidelines for the diagnosis and management of cystathionine beta-synthase deficiency. J Inherit Metab Dis. 2017 Jan;40(1):49–74.

22. Dionisi-Vici C. Deodato F. Röschinger W. Rhead W. Wilcken B. ‘Classical’ organic acidurias. propionic aciduria. methylmalonic aciduria. and isovaleric aciduria: long-term outcome and effects of expanded newborn screening using tandem mass spectrometry. J Inherit Metab Dis. 2006;29(2–3):383–9.

23. Baumgartner MR. Hörster F. Dionisi-Vici C. Haliloglu G. Karall D. Chapman KA. et al. Proposed guidelines for the diagnosis and management of methylmalonic and propionic acidemia. Orphanet J Rare Dis. 2014 Sep 2;9:130.

24. Forny P. Hörster F. Ballhausen D. Chakrapani A. Chapman KA. Dionisi-Vici C. et al. Guidelines for the diagnosis and management of methylmalonic acidaemia and propionic acidaemia: First revision. J Inherit Metab Dis. 2021 May;44(3):566–92.

25. Knerr I. Weinhold N. Vockley J. Gibson KM. Advances and challenges in the treatment of branched-chain amino/keto acid metabolic defects. J Inherit Metab Dis. 2012 Jan;35(1):29–40.

26. Joint FAO/WHO/UNU Expert Consultation on Protein and Amino Acid Requirements in Human Nutrition (2002 : Geneva S. Nations F and AO of the U. Organization WH. University UN. Protein and amino acid requirements in human nutrition : report of a joint FAO/WHO/UNU expert consultation [Internet]. World Health Organization; 2007 [cited 2024 Dec 3]. Available from: https://iris.who.int/handle/10665/43411

27. Pinto A. Evans S. Daly A. Almeida MF. Assoun M. Belanger-Quintana A. et al. Dietary practices in methylmalonic acidaemia: a European survey. J Pediatr Endocrinol Metab. 2020 Jan 28;33(1):147–55.

28. Häberle J. Burlina A. Chakrapani A. Dixon M. Karall D. Lindner M. et al. Suggested guidelines for the diagnosis and management of urea cycle disorders: First revision. J Inherit Metab Dis. 2019 Nov;42(6):1192–230.

29. Dionisi-Vici C. Rizzo C. Burlina AB. Caruso U. Sabetta G. Uziel G. et al. Inborn errors of metabolism in the Italian pediatric population: a national retrospective survey. J Pediatr. 2002 Mar;140(3):321–7.

30. Leonard JV. Morris A a. M. Urea cycle disorders. Semin Neonatol. 2002 Feb;7(1):27–35.
